# Host–Guest Induced Peptide Folding with Sequence-Specific Structural Chirality

**DOI:** 10.1021/jacs.1c00342

**Published:** 2021-04-16

**Authors:** David E. Clarke, Guanglu Wu, Ce Wu, Oren A. Scherman

**Affiliations:** Melville Laboratory for Polymer Synthesis, Department of Chemistry, University of Cambridge, Lensfield Road, Cambridge CB2 1EW, United Kingdom

## Abstract

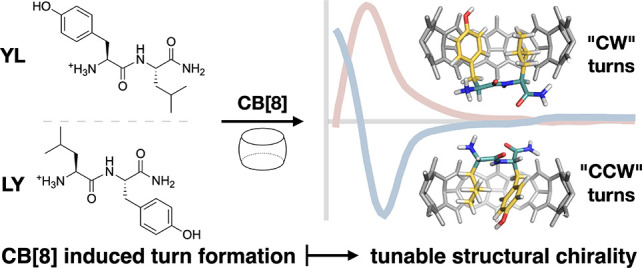

Controlling the spatial and temporal behavior of peptide segments is essential in the fabrication of functional peptide-based materials and nanostructures. To achieve a desired structure, complex sequence design is often required, coupled with the inclusion of unnatural amino acids or synthetic modifications. Herein, we investigate the structural properties of 1:1 inclusion complexes between specific oligopeptides and cucurbit[8]uril (CB[8]), inducing the formation of turns, and by alteration of the peptide sequence, tunable structural chirality. We also explore extended peptide sequence binding with CB[8], demonstrating a simple approach to construct a peptide hairpin.

Control over the spatial and temporal behavior of peptide segments is of great importance for precisely engineering hierarchical protein structures,^1,2^ inhibiting peptide and protein aggregation,^3,4^ and developing stimuli-responsive materials.^5−7^ Peptide architectures can be altered significantly by covalent modifications or through noncovalent molecular recognition.^8,9^ These approaches typically require precise sequence design, along with the inclusion of unnatural amino acids to achieve the desired structural function.^10−12^

Cucurbit[*n*]urils (CB[*n*]s, *n* = 5–8, 10) are a family of synthetic macrocycles that can bind specific amino acids with high affinity and selectivity.^13−17^ Specifically, the large cavity of CB[8] is versatile in accommodating peptide side chains and can adopt various configurations. In addition to 1:1 complexation, CB[8] can encapsulate one residue with an auxiliary guest to form a 1:1:1 heteroternary motif^18^ or bind two identical residues to form a 2:1 homoternary complex.^19,20^ Namely, the motif containing two Phe-Gly-Gly has been applied to generate protein dimers or supramolecular polymers via binding with CB[8].^21,22^

CB[8] binding motifs can be classified through their binding stoichiometry coupled with their corresponding enthalpy perturbations.^23^ Urbach and co-workers have reported 1:1 binding between CB[8] and specific tripeptide sequences such as Tyr-Leu-Ala (YLA) and Met-Leu-Ala (MLA), which exhibit abnormally large enthalpy changes similar to those found for ternary complexes.^24,25^ Through investigating their size-based diffusion properties, we elucidated that rather than forming arbitrary n:n aggregates, this unique complex contained exactly one CB[8] and one peptide.^26^ In this configuration, two adjacent side chains of the peptide simultaneously reside inside the cavity, resembling a ternary motif but with the exception that the encapsulated moieties are connected via a covalent amide bond.^24−26^ This binding motif exhibits a *host-mediated adjacent side-chain pairing* (HASP), which we have termed a “HASP knot”, representing a pseudostatic complexation tecton.

The wider applications of HASP knots are relatively unexplored, with previous studies focusing on the thermodynamic aspects of complexation.^24−26^ The main findings can be summarized as follows: (1) an adjacent residue pair from any combination of Tyr (Y), Leu (L), Lys (K), Phe (F), Met (M), and Arg (R) is inclined to form a HASP knot;^24,25^ (2) the binding affinity is more susceptible to the position of the pair (*K*_N-terminus_ > 10*K*_C-terminus_) than its sequence order (*K*_YL_ ≈ *K*_LY_); (3) although only two residues are necessary, an additional third residue or further extension in the sequence can stabilize the pair-inclusion configuration, leading to quantitative formation of HASP knots with a substantial suppression on other competitive pathways.^26^

Formation of a HASP knot requires the peptide backbone to adopt a constrained conformation, whereby the amino acid side chains rotate around the amide bond so they are located on the same side plane of the peptide. This distortion angle, referred to as the angle of rotation between the two side chains needs to approach 0° (Figure S1). In a survey of approximately 200 dipeptide crystal structures found in the CCDC database, <20% were smaller than 90° and only 10% were within 10°, with these predominantly existing in aromatic–aromatic species such as FF.^27,28^ Therefore, it is conformationally unfavorable for a dipeptide to adopt such an acute distortion angle.

Herein, we investigated the hypothesis whether HASP knots could be a feasible approach to precisely manipulate peptide conformation. We explore their structural properties focusing on dipeptide isomers (constitutional- and stereo-), leading us to the discovery of both their sequence-specific structural chirality and the amplification of their circular dichroism (CD) signals. In addition, through the design and site-specific CB[8] labeling of extended peptide sequences, we demonstrate the formation of a peptide hairpin via Förster resonance energy transfer (FRET).

Through utilizing CD spectroscopy, we identified that the formation of HASP knots can induce a significant conformational change. As shown in Figure 1b, YLA (all peptides have a C-terminal amide, unless stated otherwise) and YAL exhibit similar CD signatures with a negative minimum at 195 nm and a smaller positive inflection at 225 nm, suggesting typical random coil conformations.^29^ When complexed with CB[8] at a 1:1 ratio, the CD spectra of YLA@CB[8] (orange trace) displayed a new positive maxima at around 200 nm and a large increase in signal intensity and the inflection at 225 nm is no longer pronounced. Conversely, YAL@CB[8] (green trace) which does not form a HASP knot,^24^ has a signature similar to its uncomplexed species resembling a random coil. These significant transformations of YLA@CB[8]’s CD spectra suggest the generation of a new secondary structure arising from the formation of a HASP knot. The same spectral behavior was also observed for YMA, YKA, YRA, and FLA, which all readily form HASP knots with CB[8] (Figure 1b).^24−26^ This phenomenon is also found in dipeptide sequences such as YL@CB[8] (Figure 1c, blue trace), although slight spectral differences exist when compared to YLA@CB[8]. Therefore, to avoid unnecessary complexity introduced by Ala, which is positioned outside the CB[8] cavity, we opted to concentrate on dipeptide sequences for subsequent chirality studies.

**Figure 1:**
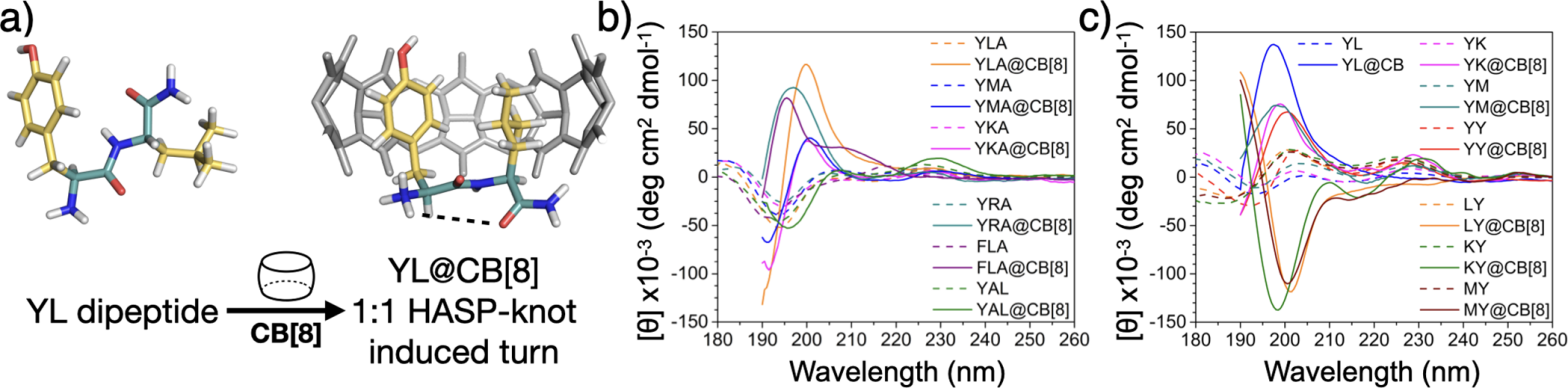
(a) Proposed conformational change of the YL dipeptide when forming a 1:1 HASP knot with CB[8]. CD spectra of tripeptides (b) and dipeptides (c) and their (1:1) CB[8] complexes.

In line with previous studies on tripeptides,^24,25^ isothermal titration calorimetry and NMR experiments revealed that Y residues coupled with an aliphatic amino acid (YL/LY, YM/MY, YK/KY) or an additional Y residue (YY) were all found to favor the formation of HASP knots (Figures S2–S9).^26^ The CD spectra of these dipeptides display similar CD signatures, with minima around 190 nm and positive inflections at 200 and 225 nm (Figure 1c). When complexed with CB[8] at a 1:1 ratio, the aromatic–aliphatic dipeptide (YM/YK@CB[8]) CD spectra both undergo similar transformations to YL@CB[8]. Interestingly, the aliphatic–aromatic dipeptides (LY/MY/KY@CB[8]) displayed different transformations to their aromatic–aliphatic counterparts, exhibiting an inverse negative minima around 195–200 nm (Figure 1c). A chiral inversion induced through swapping the sequence order of adjacent residues is highly unique and offers an approach to manipulate peptide folding in a controllable manner.

CD signals of peptide sequences in the near UV are typically generated by π → π* (190–200 nm) and *n* → π (215–230 nm) transitions of the amide bond.^30^ Therefore, the intense peaks observed around 195–200 nm in the HASP knots originate from an electronic transition from NH to a carbonyl bond (π → π*) and resemble spectra of β-turns.^31,32^ Given the acute angle required for the dipeptide to fold into the CB[8] cavity, it suggests that this signal arises from an induced π → π* transition present in the complex’s constrained conformation (Figure 1a). This is further supported by the CD spectra of YL at different ratios of CB[8], where both the evolution and saturation of the positive maxima can be visualized (Figure S10).

CD spectra of the aromatic–aromatic dipeptides (FF and YY) already display a small increase in signal intensity with larger inflections at both 190 and 230 nm, along with a positive maxima around 200 nm (Figure S11a). Hyperchromism is often attributed to the formation of higher order secondary structures such as β-sheets and β-turns,^30,32^ which has been observed for FF.^33^ This effect is typically due to the ordering of chromophores into arrays (β-sheet) or localized structures (β-turn), where the lowest energy transitions become hyperchromic.^30^ Previous reports suggest FF already adopts a β-turn-like structure in aqueous media, with an acute angle existing between the dipeptide’s side chains.^27,33^ Given the spectral and structural similarities between YY and FF, it is likely that YY also has a β-turn-like conformation in solution (Figure S11a). Upon complexation, the YY@CB[8] HASP knot retains similar spectral features to YY with the same 230 nm inflection and a smaller signal increase in the 200 nm positive maxima. This suggests that a similar spatial structure and electronic transitions are maintained (Figures 1c and S11b). These observations infer the side chains in YL/YM/YK@CB[8] HASP knots are rotated to the same side-plane of the peptide backbone forming a “clockwise” (CW) turn, a structure akin to YY@CB[8] (ψ_1_ ≈ 155°, ϕ_2_ ≈ 55°) (Figure 2a). This is further supported by the hyperchromism displayed for these complexes, where the induced turn permits a π → π* transition of lower energy. For the aliphatic–aromatic dipeptides (LY/MY/KY@CB[8]), the inverse chiral signals imply a turn is generated but in the opposite “counterclockwise” (CCW) direction (ψ_1_ ≈ −125°, ϕ_2_ ≈ −50°) (Figure 2b).

**Figure 2:**
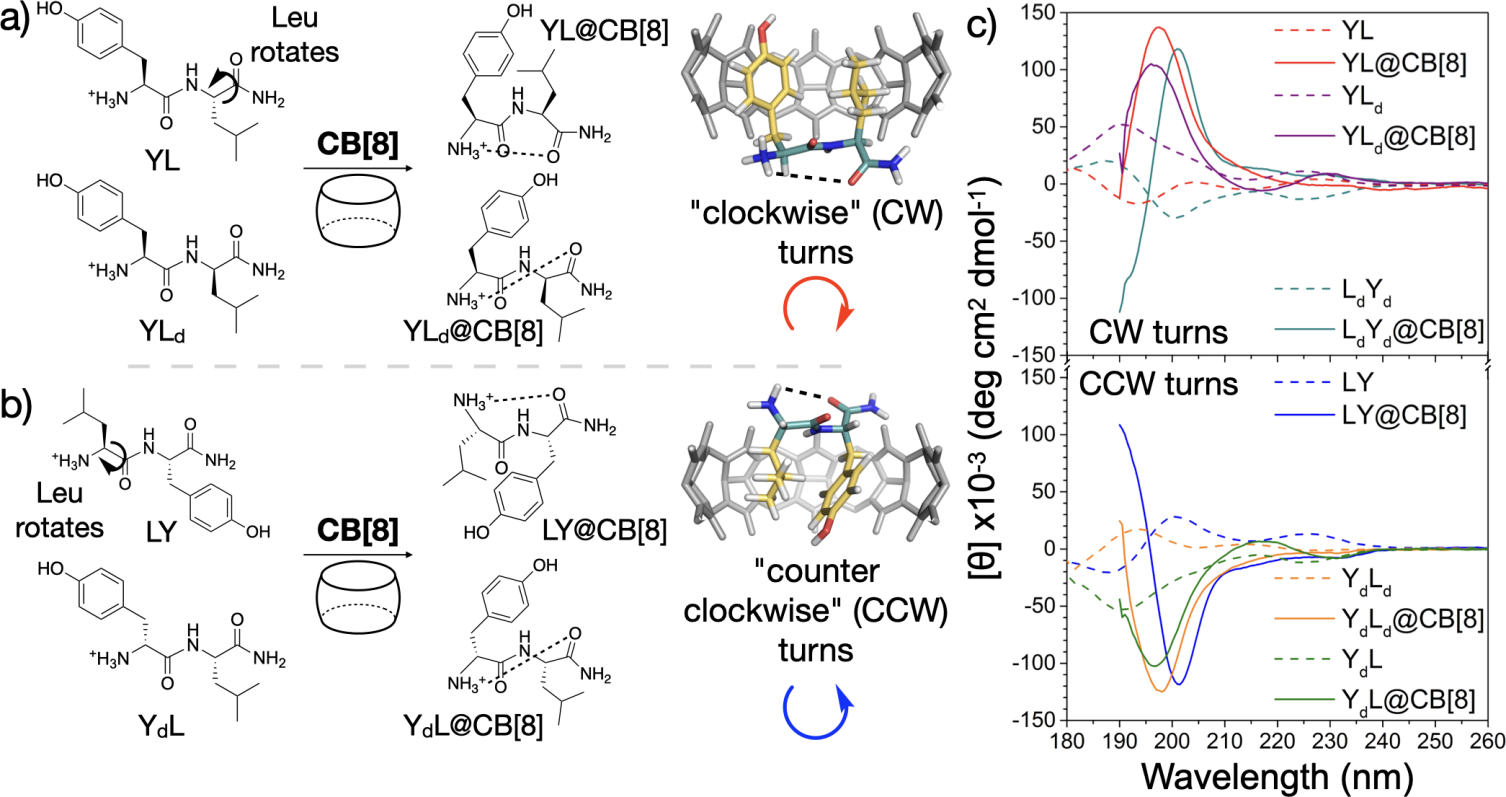
(a) “Clockwise” turns are generated through Leu rotation in YL@CB[8] (top) or via d-Leu in YL_*d*_@CB[8] (bottom). (b) “Counterclockwise” turns are formed through Leu rotation in LY@CB[8] (top) or via d-Tyr in Y_*d*_L@CB[8] (bottom). (c) CD spectra of Tyr and Leu containing dipeptides and their CB[8] complexes, highlighting CW (top) or CCW (bottom) turn formation.

To further investigate the chiral transitions of HASP knots, we synthesized a variety of dipeptides with combinations of l- and d-amino acids (based on Y and L). NMR studies confirmed the formation of HASP knots for all constitutional and stereoisomers of YL (Figures S15, S18, S19 and S22). The d-isomers of YL (Y_*d*_L_*d*_) and LY (L_*d*_Y_*d*_) had CD spectra that mirror those of the natural l-isomers both before and after complexation with CB[8] (Figures 2c, S12, and S13). Therefore, both chiral isomers form opposite turns when encapsulated inside the CB[8] host, where YL/L_*d*_Y_*d*_ (CW) and LY/Y_*d*_L_*d*_ (CCW) are in the same direction, respectively.

Heterochiral sequences of YL containing both l- and d-amino acids were also investigated. The side chains of Y_*d*_L and YL_*d*_ already reside on the same side of the amide bond but are in opposite directions with respect to each other (Figure 2a and b). This was verified by the mirrored CD signatures obtained for Y_*d*_L and YL_*d*_ (Figures 2c and S14a). These unnatural heterodipeptides inherently have an acute angle between side chains as they reside on the same side of the amide bond. With this in mind, HASP knot formation with CB[8] resulted in a similar spatial conformation being retained through complexation (Figure 2a and b). However, since Y_*d*_L and YL_*d*_ are chiral isomers, the CB[8] induced turns are generated in opposite directions and this can be visualized in their CD spectra (Figures 2c and S14b). These isomeric complexes suggest YL@CB[8]/YL_*d*_@CB[8] (CW) and LY@CB[8]/Y_*d*_L@CB[8] (CCW) adopt similar structures. Therefore, in both the YL@CB[8] and LY@CB[8] complexes, we can suggest the L side chain rotates toward the Y residue during complexation to form turns that are opposite in direction (Figure 2a and b).

We validated our model by performing NOESY measurements on the Y_*d*_L_*d*_ and YL_*d*_ dipeptides. When uncomplexed, Y_*d*_L_*d*_ has no detectable correlation between side chains, confirming they reside on opposite sides of the amide bond (Figure S20). Following HASP knot formation with CB[8], NOE correlations could be recognized between the two adjacent side chains, which suggests rotation around the amide bond and the formation of a turn-like structure (Figure S21). In contrast, different behavior is observed for YL_*d*_ where NOE correlations between side chains are present both before and after complexation (Figures S16 and S17). This provides further evidence for our hypothesis, where the side chains of YL_*d*_ remain on the same side of the amide bond and following HASP knot formation, adopt a turn conformation. These CD and NMR studies demonstrate that simple changes in the ordering of a peptide sequence can be utilized to control a localized chiral environment and the corresponding direction of the induced turn.

To apply our findings, we investigated whether HASP knots could be applied to extended peptide sequences as an elementary operation to manipulate their spatial structure. We rationally designed the sequence YLAGGAFLAGGALY, which has three binding sites: FL (midchain), YL (N-terminus), and LY (C-terminus) (Figure S23). These three HASPs were coupled with neighboring Ala residues, which have been reported to stabilize pair inclusion complexes.^26^ In the presence of excess CB[8] (1:3.6 peptide:CB[8]), the NMR spectrum of YLAGGAFLAGGALY is almost identical to the superposition of the spectra of YLA@CB[8], AFLA@CB[8], and ALY@CB[8] (Figure S23). Diffusion ordered spectroscopy (DOSY) NMR measurements also displayed a diffusion value corresponding to a complex containing three CB[8]s (Figure S24 and Table S2),^34^ confirming that HASP knots can be generated independently at multiple sites and are not restricted to the peptide’s termini.

To demonstrate that a hairpin structure can be induced by the formation of a HASP knot, we designed a sequence WGGAYLAGG-Dansyl (h-peptide) containing a FRET pair of Trp (W) and a Dansyl fluorophore positioned at opposite termini, coupled with a HASP (-AYLA-) midchain binding-site. NMR of the h-peptide at different ratios of CB[8] displayed broad peaks and indistinguishable binding modes until an excess of CB[8] was reached (Figures S26). At a ratio of 1:6 (h-peptide:CB[8]), ^1^H and DOSY NMR showed that a HASP knot formed at the -AYLA- site accompanying 1:1 CB[8] binding of the W and Dansyl residues (Figures S27, S28 and Table S2). Considering that both Dansyl and W are each independently encapsulated by CB[8] macrocycles, only long-range energy transfer through space (e.g., FRET) is permitted between these two chromophores.

Steady-state emission spectra were recorded for the h-peptide sequence before and after the addition of CB[8] (Figure 3b). Without CB[8], two emission bands were observed at 360 and 560 nm (λ_ex_ 225 nm) corresponding to the photoluminescent emissions of W and Dansyl, respectively. In the presence of excess CB[8] (1:6 h-peptide:CB[8]), the emission band of W diminishes by 70% and is coupled with a 2.3 fold increase in Dansyl emission (Figure 3b). This observed FRET indicates the close proximity of the N- and C- termini and confirms the formation of a hairpin structure induced by a HASP knot.

**Figure 3:**
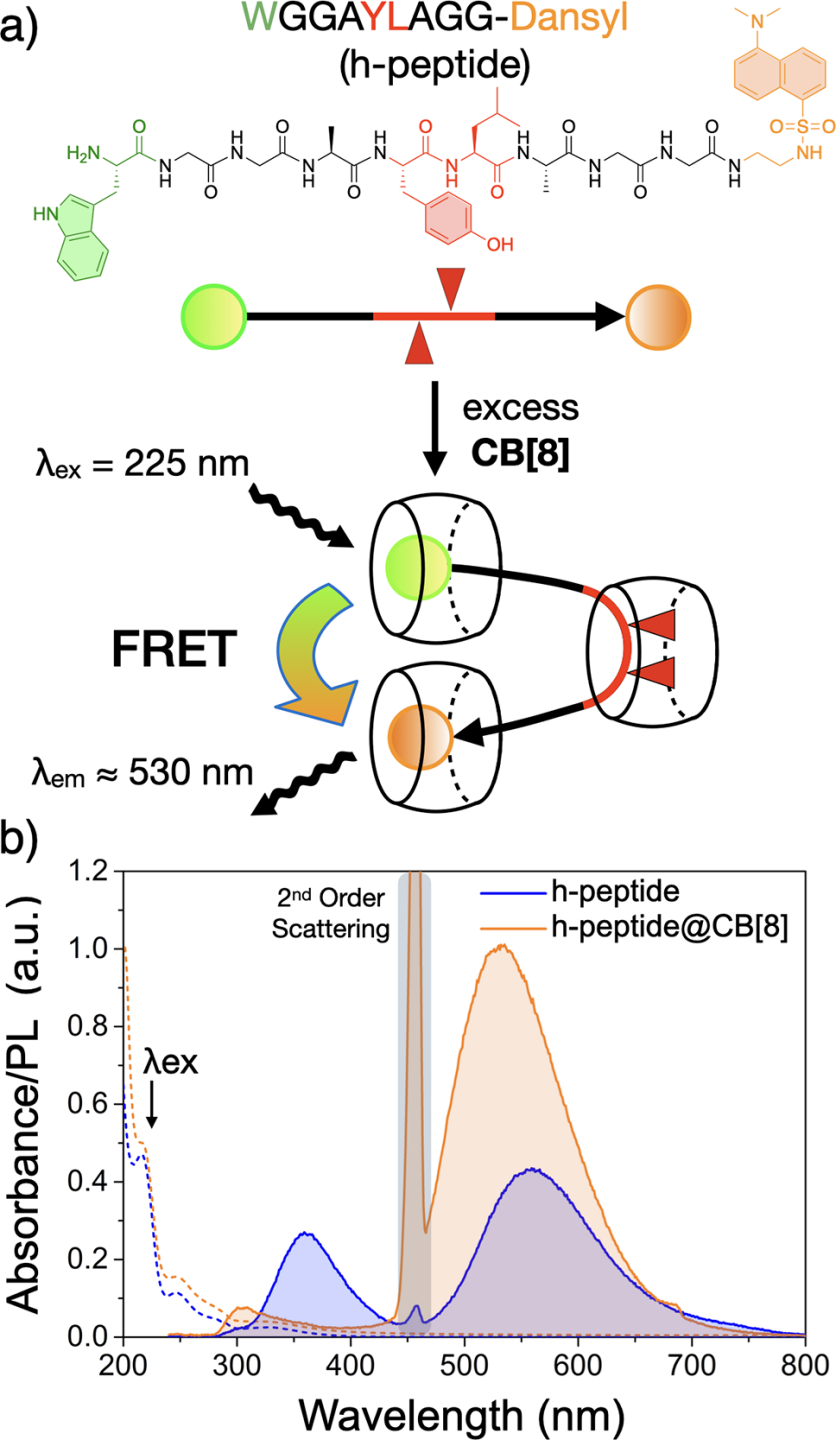
(a) A hairpin structure is generated through h-peptide complexation with CB[8]. (b) Steady-state spectra for the h-peptide and its CB[8] complex, highlighting hairpin formation via FRET (λ_*ex*_ 225 nm). All measurements were performed in 15 mM Na_2_CO_3_ buffer. Dashed lines represent corresponding absorbance spectra.

Through investigating the structural chirality of dipeptides and their CB[8]-mediated adjacent side-chain pairing, i.e., HASP knot, we discovered the formation of an induced turn-like structure. Interestingly, by altering the ordering of the peptide sequence or the inclusion of unnatural amino acids, the direction of the turn can be manipulated. This sequence-specific structural chirality could be incredibly powerful in tuning localized chiral environments for asymmetric catalysis. We also explored how HASP motifs can be applied to extended peptide sequences as a simple operation to influence their wider structure. By combining midsequence binding and the ability to induce a turn, we utilized a peptide sequence containing a FRET pair to confirm that CB[8] binding can induce the formation of a peptide hairpin. This work provides a simple approach to manipulate peptide segments, where mutations containing HASP motifs can be introduced into intrinsically disordered regions of proteins to provide structural function. Moreover, there is potential for this approach to be explored in the design and fabrication of chiral supramolecular complexes and other engineered nanostructures.

## Supporting Information Available

Materials and methods; synthesis and characterization of peptide@CB[8] complexes; isothermal titration thermograms and thermodynamic data of CB[8]-mediated complexes; circular dichroism spectra of peptide@CB[8] complexes; ^1^H NMR titrations of peptides into CB[8]; NOESY 2D NMR of YL_*d*_ and Y_*d*_L_*d*_ before and after addition of CB[8]; DOSY 2D NMR of YLAGGAFLAGGALY@CB[8] and h-peptide@CB[8] complexes; UV/vis of the h-peptide@CB[8] complex (PDF)
