# Supplementary material for: Host–Guest Induced Peptide Folding with Sequence-Specific Structural Chirality
Source: J Am Chem Soc. 2021 Apr 16;143(17):6323–7. doi: 10.1021/jacs.1c00342 (PMC8154536; doi:10.1021/jacs.1c00342)
Supplement: Supplementary file 1 — ja1c00342_si_001.pdf [file ja1c00342_si_001.pdf]

Supporting Information

Host-Guest Induced Peptide Folding with Sequence-Specific Structural Chirality

David E. Clarke,† Guanglu Wu,† Ce Wu, and Oren A. Scherman*

Melville Laboratory for Polymer Synthesis, Department of Chemistry, University of Cambridge, Lensfield Road, Cambridge CB2 1EW, UK.

* E-mail: oas23@cam.ac.uk

† These authors contributed equally to this work

Table of Contents

SI-1 Materials and methods 1
SI-2 Isothermal titration thermograms of peptides with CB[8] 4
SI-3 Circular dichroism spectra of peptides and their CB[8] complexes. 9
SI-4 NMR of peptides and their complexation with CB[8] 12
SI-5 UV/Vis of h-peptide and its CB[8] complex 21
Reference: 21

SI-1 Materials and methods

Chemical Structures of Studied Molecules.

Tripeptides

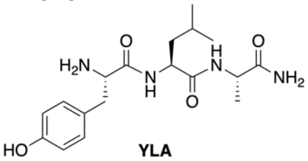

YLA

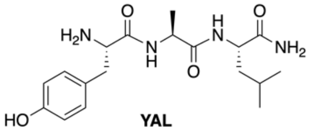

YAL

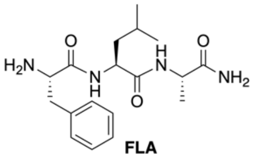

FLA

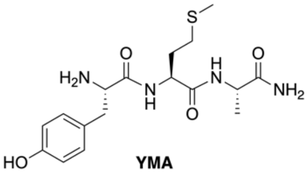

YMA

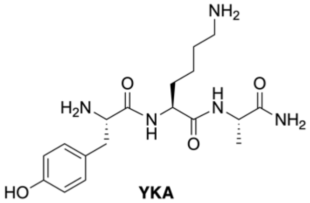

YKA

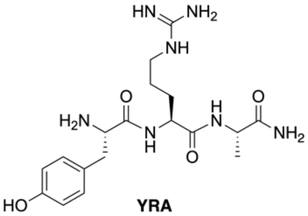

YRA

Natural Dipeptides

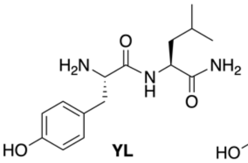

YL

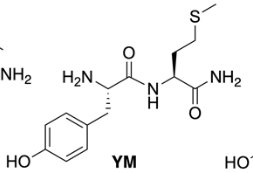

YM

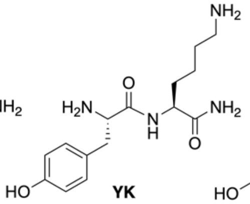

YK

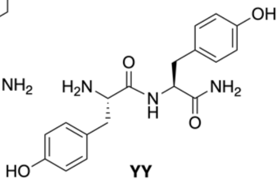

YY

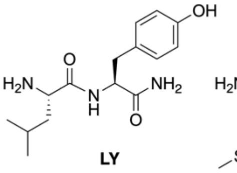

LY

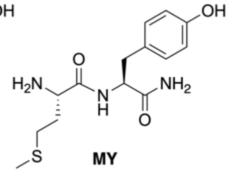

MY

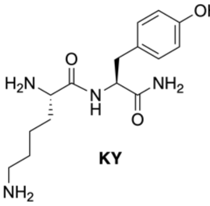

KY

Unnatural Dipeptides

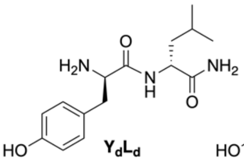

Y_dL_d

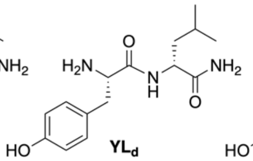

YL_d

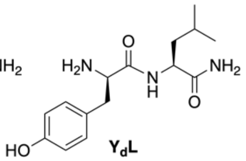

Y_dL

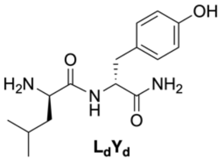

L_dY_d

Extended Peptide Sequences

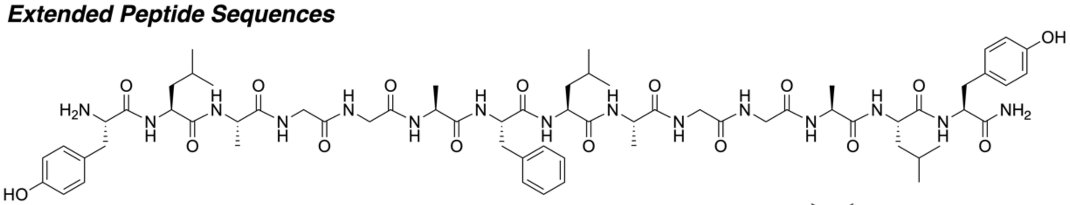

YLAGGAFLAGGALY

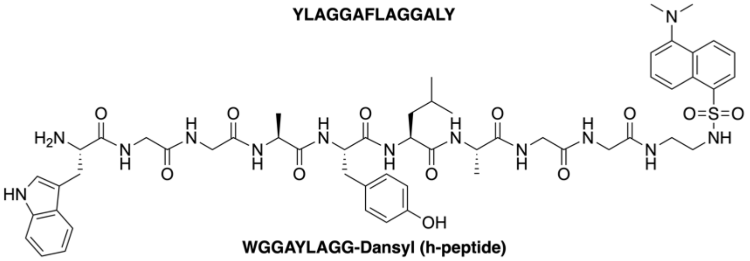

WGGAYLAGG-Dansyl (h-peptide)

Materials. All Fmoc-protected amino acids, solvents, and rink amide 4-methyl-benzhydrylamine (MBHA) resin used for peptide synthesis were purchased from AGTC Bioproducts (UK), except for Dansyl-NovaTag^{TM} resin which was purchased from Sigma-Aldrich (UK). All other solvents and reagents were purchased from Sigma-Aldrich (UK) and used as received. Cucurbit[8]uril (CB[8]) was synthesized according to the published procedure [1]. Milli-Q water (18.2 MΩ·cm) was used for preparation of all non-deuterated aqueous solutions. The stock solution of 10 mM sodium phosphate buffer was prepared by mixing sodium phosphate monobasic, sodium phosphate dibasic, and water then adjusting to pH 7.0.

Peptide Synthesis and Characterisation. All peptide sequences were synthesized using solid-phase methodology (FMOC, tBu, MBHA or Dansyl-NovaTag^{TM} resin) on an automated microwave peptide synthesiser (LibertyBlue, CEM). Crude Peptides were cleaved from the resin with a cleavage cocktail of 95% trifluoroacetic acid (TFA), 2.5% triisopropyl silane and 2.5% DI H_2O and left to shake for 2.5 h. Following cleavage, the crude peptides were precipitated and washed with cold diethyl ether (DEE), then left to dry under vacuum overnight.

The crude peptides were then purified by high pressure liquid chromatography (HPLC) using a Phenomenex C18 Kinetic-Evo column with a 5 µm pore size, a 110 Å particle size and with the dimensions 150 x 21.2 mm. A gradient from 5% acetonitrile 95% water to 100% acetonitrile was run with 0.1% TFA. Following purification, peptide identities were verified by analytical HPLC and ^{1}H-NMR.

Isothermal Titration Calorimetry (ITC). All ITC experiments were carried out on a Microcal iTC200 at 298.15 K in 10 mM sodium phosphate buffer (pH = 7.0). In a typical ITC, the host molecule (CB[8]) was in the sample cell, and guest molecule was in the injection syringe with a concentration of about ten times concentration of host. The concentration of CB[8] was calibrated by the titration with a standard solution of 1-adamantanamine. In order to avoid bias or potentially arbitrary offsets caused by manual adjustment of baseline, all raw data (thermograms) of ITC were integrated by NITPIC (v.1.2.0), fitted in Sedphat (v.12.1b), and visualized through GUSSI (v.1.1.0) [2]. For each species, at least two individual titrations were performed for the subsequent global fitting, whose error estimations were carried out by F statistics at the 0.68 confidence level.

Nuclear Magnetic Resonance Spectroscopy (NMR). ^{1}H NMR, ^{1}H-^{1}H NOESY, and ^{1}H DOSY spectra were acquired in heavy water (D_2O) at 298 K and recorded on a Bruker AVANCE 500 with TCI Cryoprobe system (500 MHz) being controlled by TopSpin2.

The ^{1}H DOSY experiments were carried out using a modified version of the Bruker sequence ledbpgp2s involving, typically, 32 scan over 16 steps of gradient variation from 10% to 80% of the maximum gradient. Diffusion coefficients were evaluated in Dynamic Centre (a standard Bruker software) and determined by fitting the intensity decays according to the following equation:

$$I = I_o \, e^{[-D\gamma^2 g^2 \delta^2 (\Delta - \delta/3)]}$$

where I and I_o represent the signal intensities in the presence and absence of gradient pulses respectively,

D is the diffusion coefficient, γ = 26753 rad/s/Gauss is the ^{1}H gyromagnetic ratio, δ = 2.4 ms is duration of the gradient pulse, Δ = 100 ms is the total diffusion time and g is the applied gradient strength. Monte Carlo simulation method is used for the error estimation of fitting parameters with a confidence level of 95%.

Circular Dichroism (CD). CD measurements were carried out in the far-UV on a Chirascan spectrometer (Applied Photophysics). All scans were performed at 298 K from 180-260 nm at 0.5 nm intervals with an acquisition time of 1 second and using a 0.1 cm path length quartz plate cuvette. To ensure precise concentrations for analysis, the peptide samples were directly calibrated to ratios of CB[8] following the NMR titration studies. The spectra collected was averaged across two scans and corrected via background subtraction.

UV/Vis Spectrometry. UV/Vis spectra for WGGAYLAGG-Dansyl (h-peptide) and its CB[8] complex, were recorded on a Varian Cary 4000 UV/Vis spectrophotometer using a Hellma 114F-QS cuvette with 10x4 mm path length at 298K (Figure S29). Peptide solutions were prepared at 23 μM in either D_2O (unbuffered) or with 15 mM Na_2CO_3 (buffered), and mixed with excess CB[8] (h-peptide:CB[8] 1:6).

Fluorescence Spectrometry. The fluorescence of the WGGAYLAGG-Dansyl peptide was recorded using steady-state photoluminescence emission spectra at room temperature with a Varian Cary Eclipse Flourimeter. Peptide solutions were prepared at 23 μM in D_2O with 15 mM Na_2CO_3 (± 6 equivalence of CB[8]), and the spectra recorded using a 120 μL quartz three-window fluorescence cuvette. Samples were excited at 225 nm with a 5 nm excitation/emission slit.

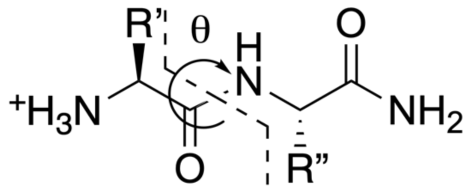

Figure S1. Schematic representing the definition of the torsion angle (θ) in dipeptides.

SI-2 Isothermal titration thermograms of peptides with CB[8]

ITC data can supply complexation information including the binding stoichiometry, enthalpy changes (dH), and the binding constant (K_a), which can further deduce Gibbs free energy changes (dG) and entropy changes (dS) through dG=-RTlnK_a=dH-TdS, where R is the gas constant and T is the absolute temperature.

Isothermal titration thermograms of the complexation between CB[8] and relevant peptides used in this study (Figure S2-S9). Most titration curves were perfectly fitted by hetero association model (AB model or one-site model). Titration curves of ,mm.;YAL were fitted through stoichiometric model (AB_2 model or sequential binding model).[3] All the data is obtained at 298.15K in 10 mM sodium phosphate buffer pH 7.0 (NaP7). Each of the thermodynamic data were obtained by the global fitting of at least two repeating experiments.

Table S1. Thermodynamic data for the association of CB[8] and the peptides used in this study

Peptides	Model	Ka AB: M^{-1}, AB2: M^{-2}	dG kcal/mol	dH kcal/mol	TdS kcal/mol	Temp. K	Buffer
YLA[3]	AB	8.1×10^6	-9.4±0.1	-12.0±0.1	-2.6±0.2	298.15	NaP7
YAL[3]	AB	2.7×10^4	-6.0±0.1	-6.2±0.6	-0.2±0.7	298.15	NaP7
YAL[3]	AB2	8.7×10^7	-10.8±0.4	-18.1±2.8	-7.3±3.2	298.15	NaP7
YMA	AB	1.0×10^6	-8.2±0.1	-11.0±0.1	-2.8±0.1	298.15	NaP7
YKA	AB	3.1×10^6	-8.9±0.1	-11.4±0.1	-2.6±0.1	298.15	NaP7
YRA	AB	1.6×10^6	-8.5±0.1	-11.1±0.1	-2.7±0.1	298.15	NaP7
FLA[3]	AB	1.0×10^7	-9.6±0.1	-12.0±0.1	-2.4±0.1	298.15	NaP7
YL[3]	AB	8.2×10^6	-9.4±0.1	-13.6±0.1	-4.2±0.2	298.15	NaP7
YM	AB	6.1×10^5	-7.9±0.1	-12.3±0.1	-4.4±0.2	298.15	NaP7
YK	AB	2.5×10^6	-8.7±0.1	-12.7±0.2	-4.0±0.2	298.15	NaP7
YY	AB	8.9×10^5	-8.1±0.1	-11.6±0.2	-3.5±0.2	298.15	NaP7
LY[3]	AB	1.3×10^7	-9.7±0.2	-13.7±0.2	-4.0±0.4	298.15	NaP7
MY	AB	3.9×10^6	-8.9±0.1	-19.2±0.2	-10.2±0.3	298.15	NaP7
KY	AB	2.4×10^6	-8.7±0.1	-11.8±0.1	-3.0±0.2	298.15	NaP7

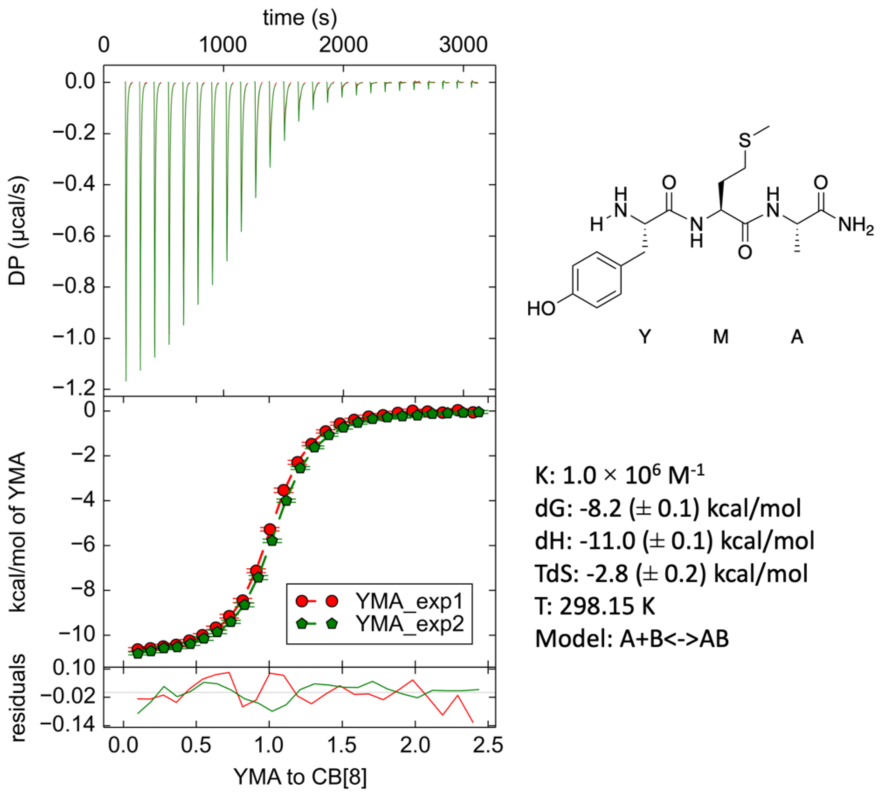

Figure S2. ITC of YMA titrated into CB[8] (0.053 mM). 10 mM sodium phosphate buffer (pH 7.0)., 298.15 K, iTC200, 25 injections of 1.5 µL, 120 s interval.

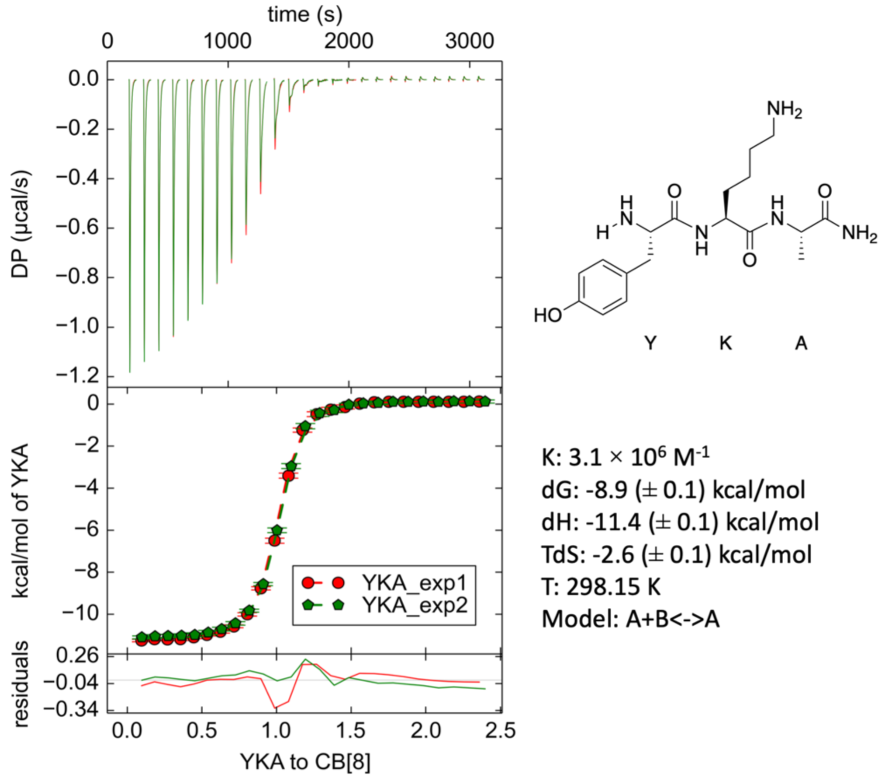

Figure S3. ITC of YKA titrated into CB[8] (0.053 mM). 10 mM sodium phosphate buffer (pH 7.0)., 298.15 K, iTC200, 25 injections of 1.5 µL, 120 s interval.

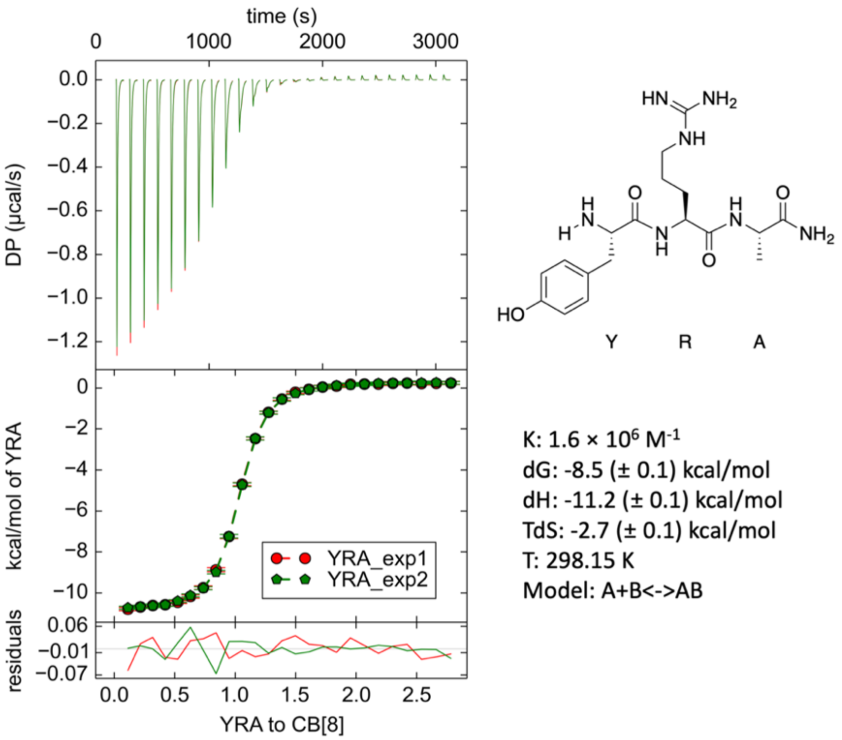

Figure S4. ITC of YRA titrated into CB[8] (0.053 mM). 10 mM sodium phosphate buffer (pH 7.0)., 298.15 K, iTC200, 25 injections of 1.5 µL, 120 s interval.

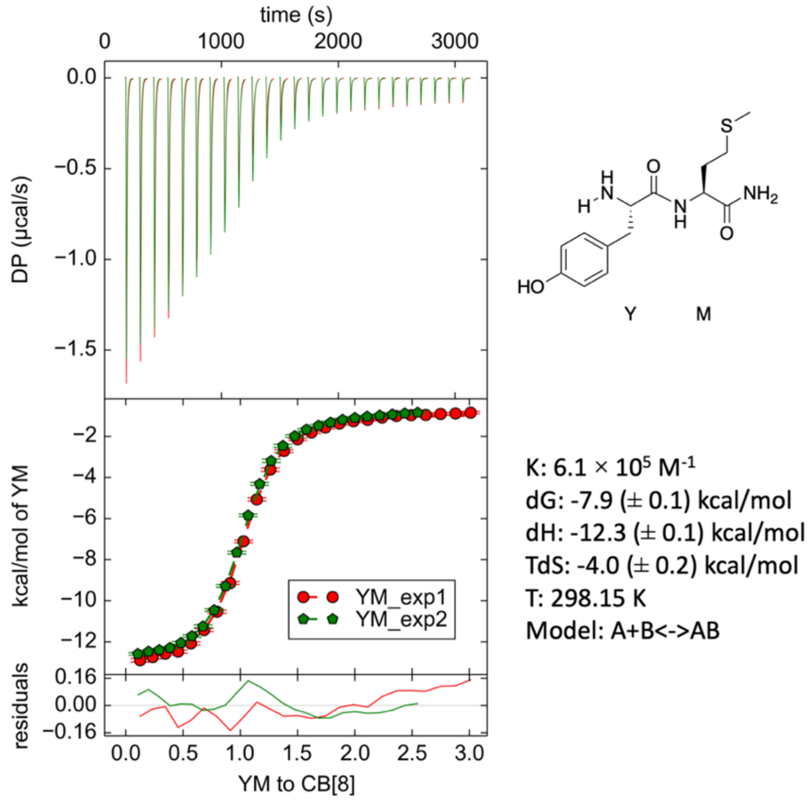

Figure S5. ITC of YM titrated into CB[8] (0.053 mM). 10 mM sodium phosphate buffer (pH 7.0)., 298.15 K, iTC200, 25 injections of 1.5 µL, 120 s interval.

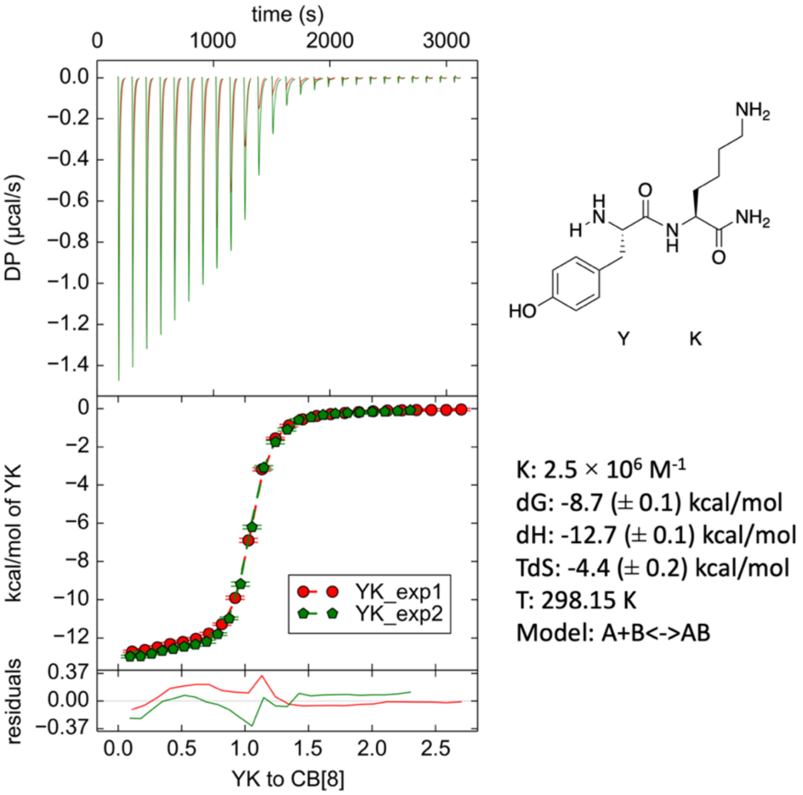

Figure S6. ITC of YK titrated into CB[8] (0.053 mM). 10 mM sodium phosphate buffer (pH 7.0)., 298.15 K, iTC200, 25 injections of 1.5 μL, 120 s interval.

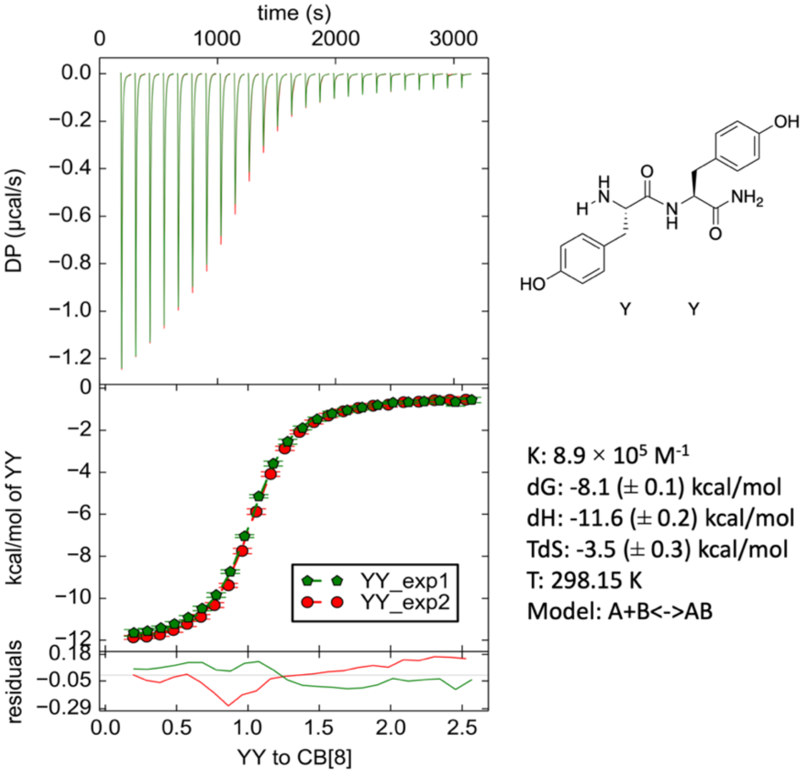

Figure S7. ITC of YY titrated into CB[8] (0.053 mM). 10 mM sodium phosphate buffer (pH 7.0)., 298.15 K, iTC200, 25 injections of 1.5 μL, 120 s interval.

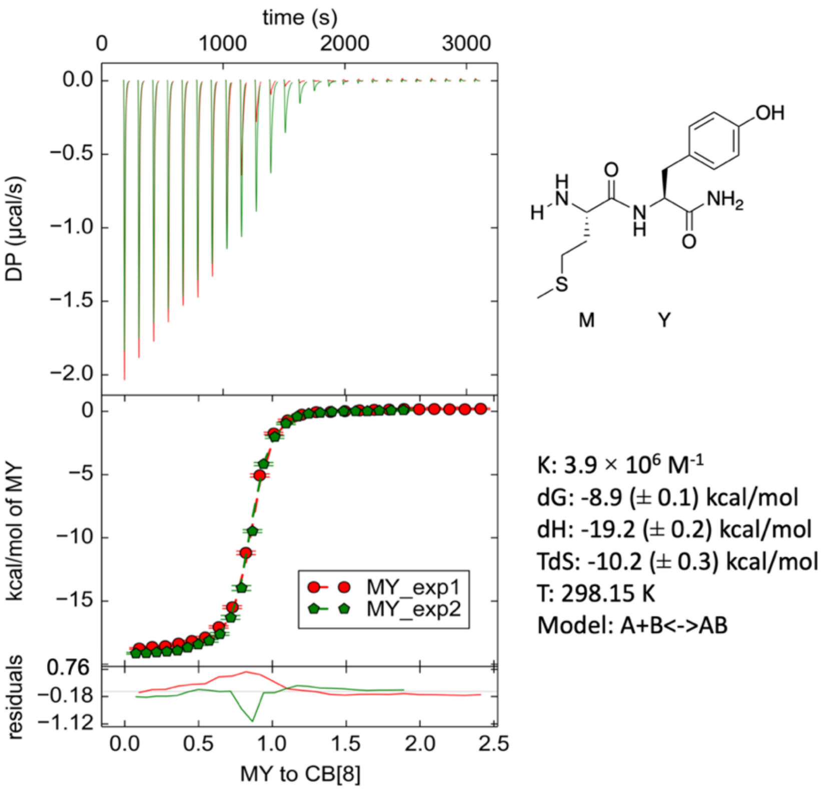

Figure S8. ITC of MY titrated into CB[8] (0.0587 mM). 10 mM sodium phosphate buffer (pH 7.0)., 298.15 K, iTC200, 25 injections of 1.5 µL, 120 s interval.

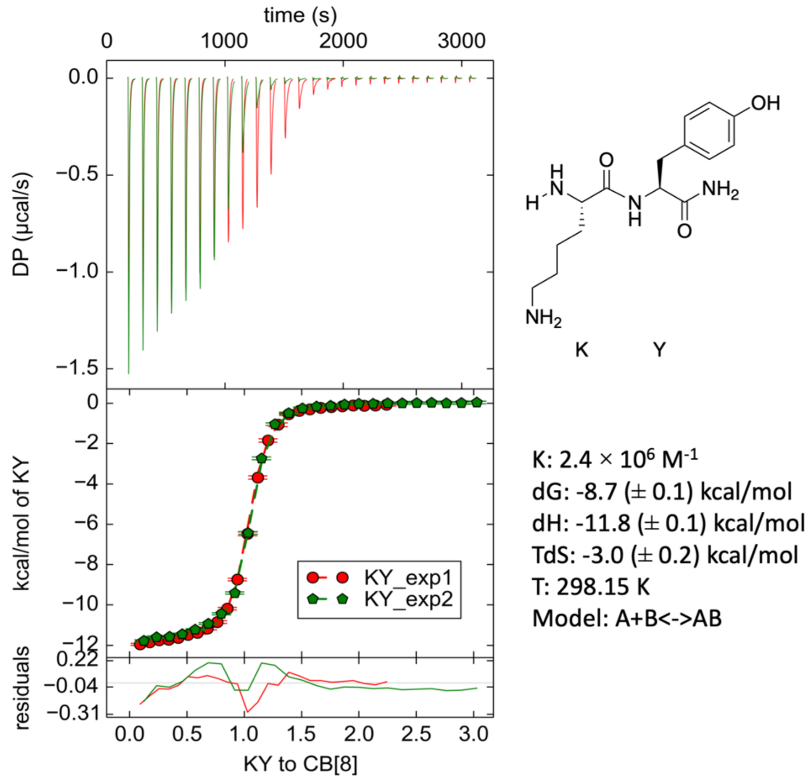

Figure S9. ITC of KY titrated into CB[8] (0.0587 mM). 10 mM sodium phosphate buffer (pH 7.0)., 298.15 K, iTC200, 25 injections of 1.5 µL, 120 s interval.

SI-3 Circular dichroism spectra of peptides and their CB[8] complexes.

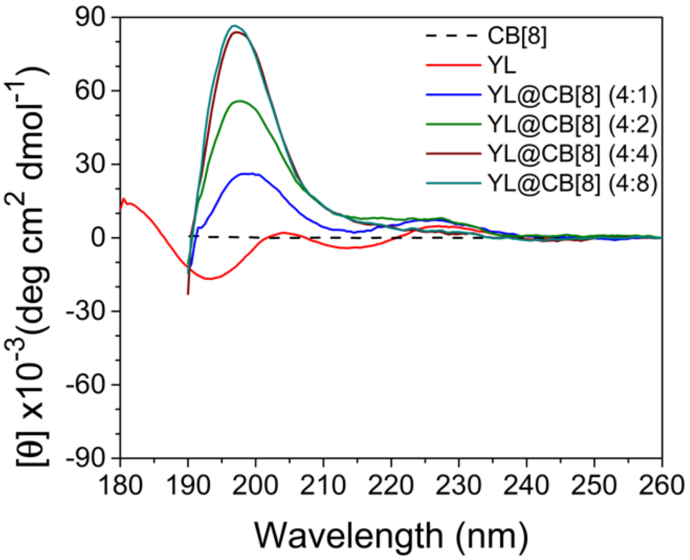

Figure S10. Circular Dichroism spectra of the YL dipeptide at different ratios of CB[8].

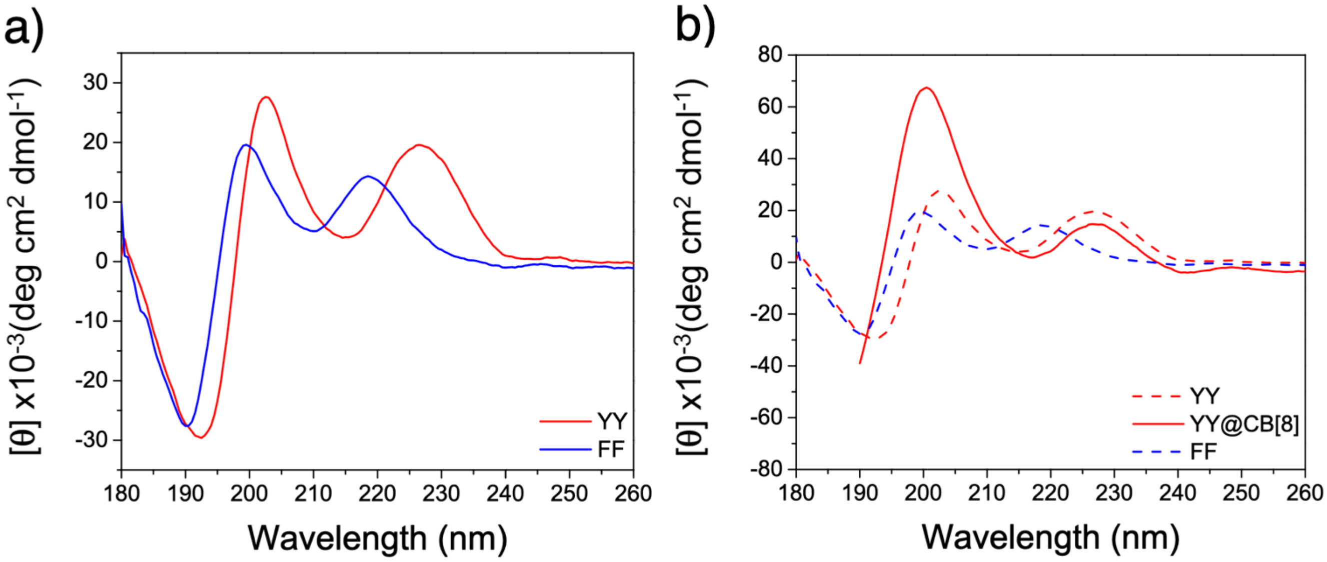

Figure S11. Circular Dichroism spectra of the YY, FF dipeptides (a), and YY@CB[8] (1:1) (b).

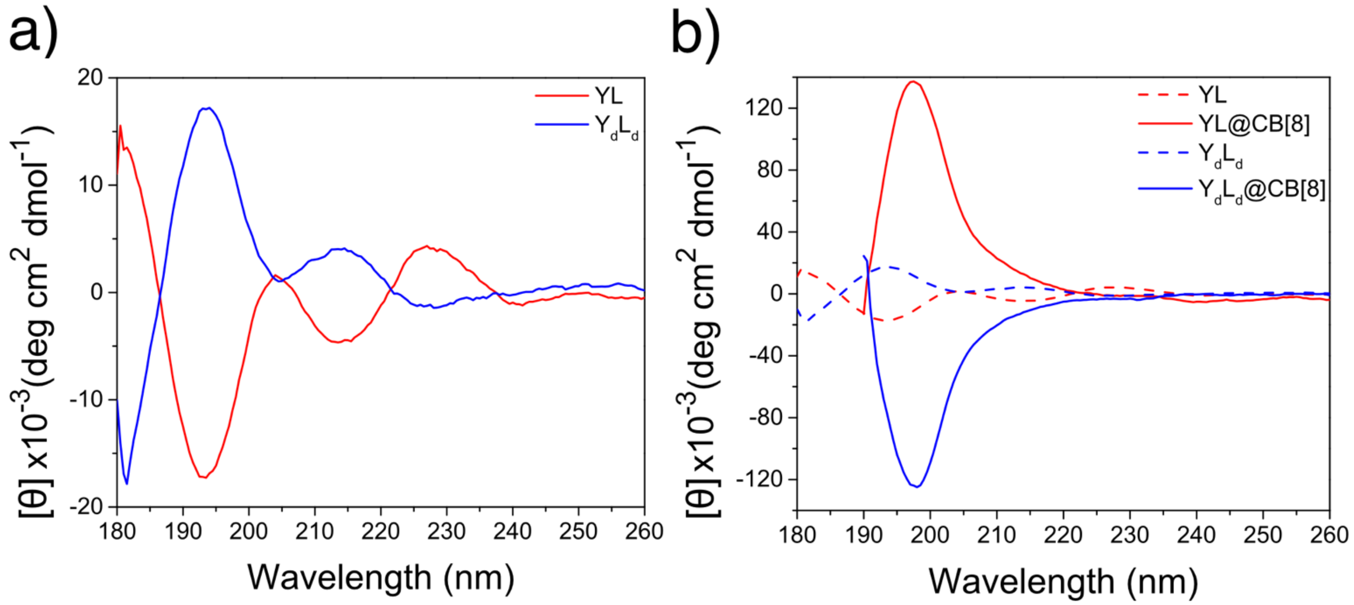

Figure S12. Circular Dichroism spectra of the YL and Y_dL_d dipeptides (a), and their (1:1) complexes with CB[8] (b).

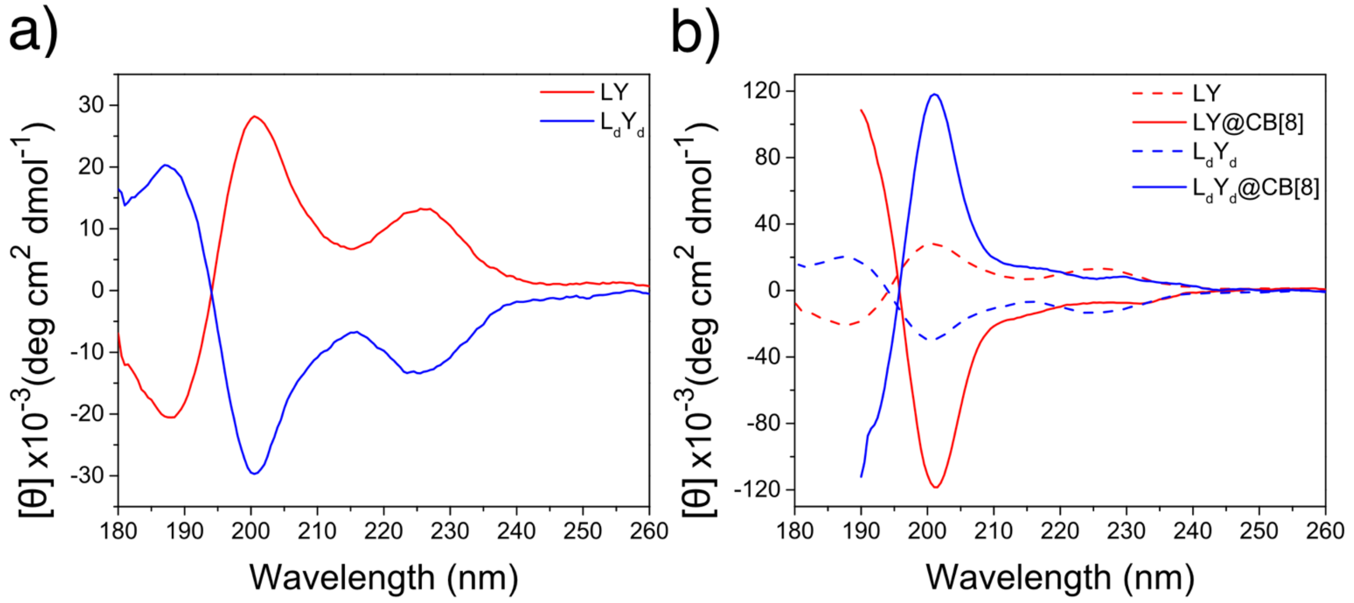

Figure S13. Circular Dichroism spectra of the LY and L_dY_d dipeptides (a), and their (1:1) complexes with CB[8] (b).

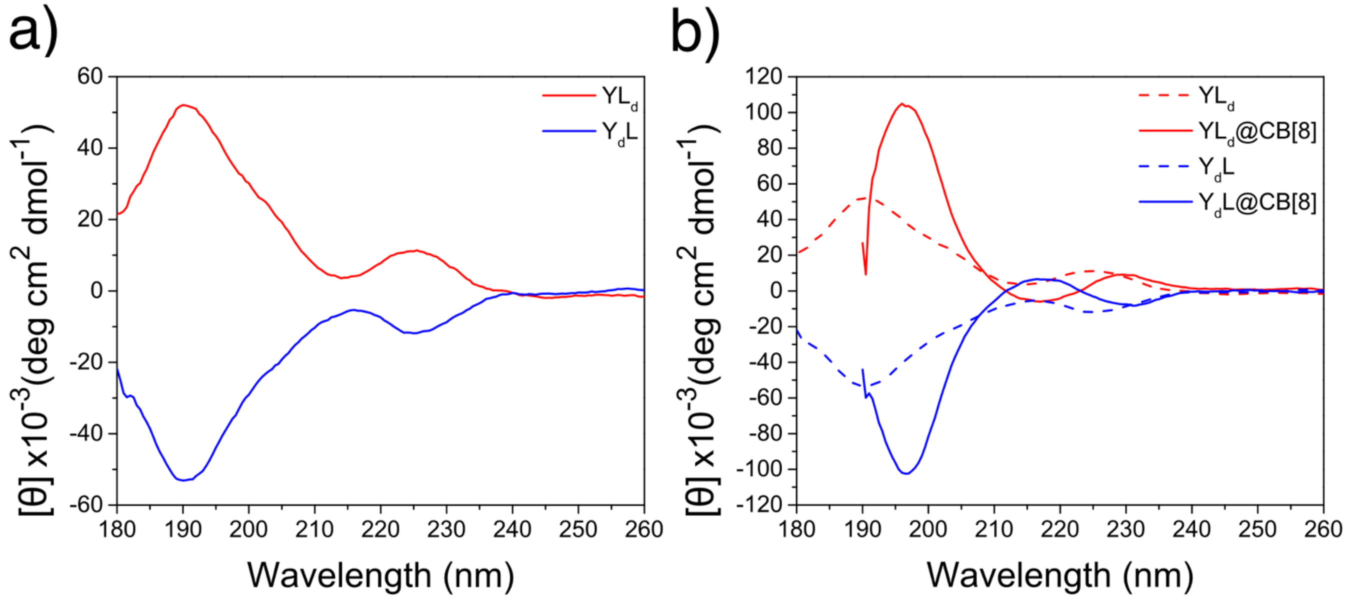

Figure S14. Circular Dichroism spectra of the YL_d and Y_dL dipeptides (a), and their (1:1) complexes with CB[8] (b).

SI-4 NMR of peptides and their complexation with CB[8]

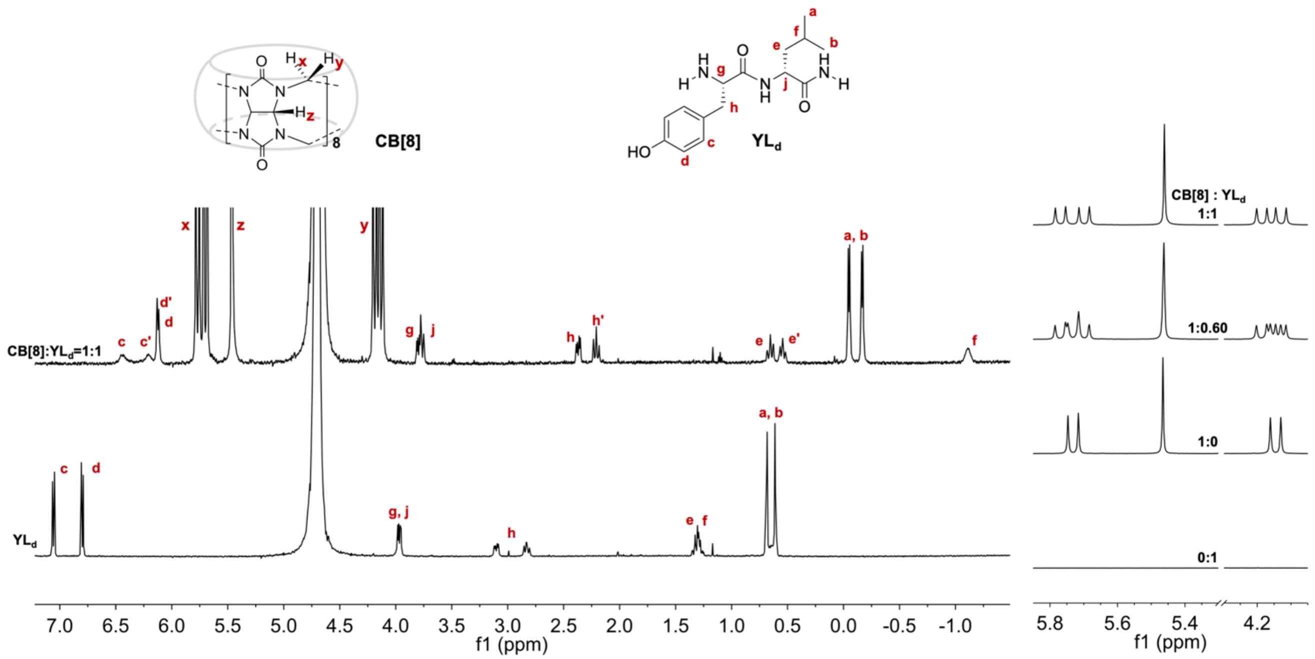

Figure S15. ^{1}H NMR of YL$_d$ titrated into CB[8] until a ratio of 1:1. (500 MHz, 298 K, D_2O).

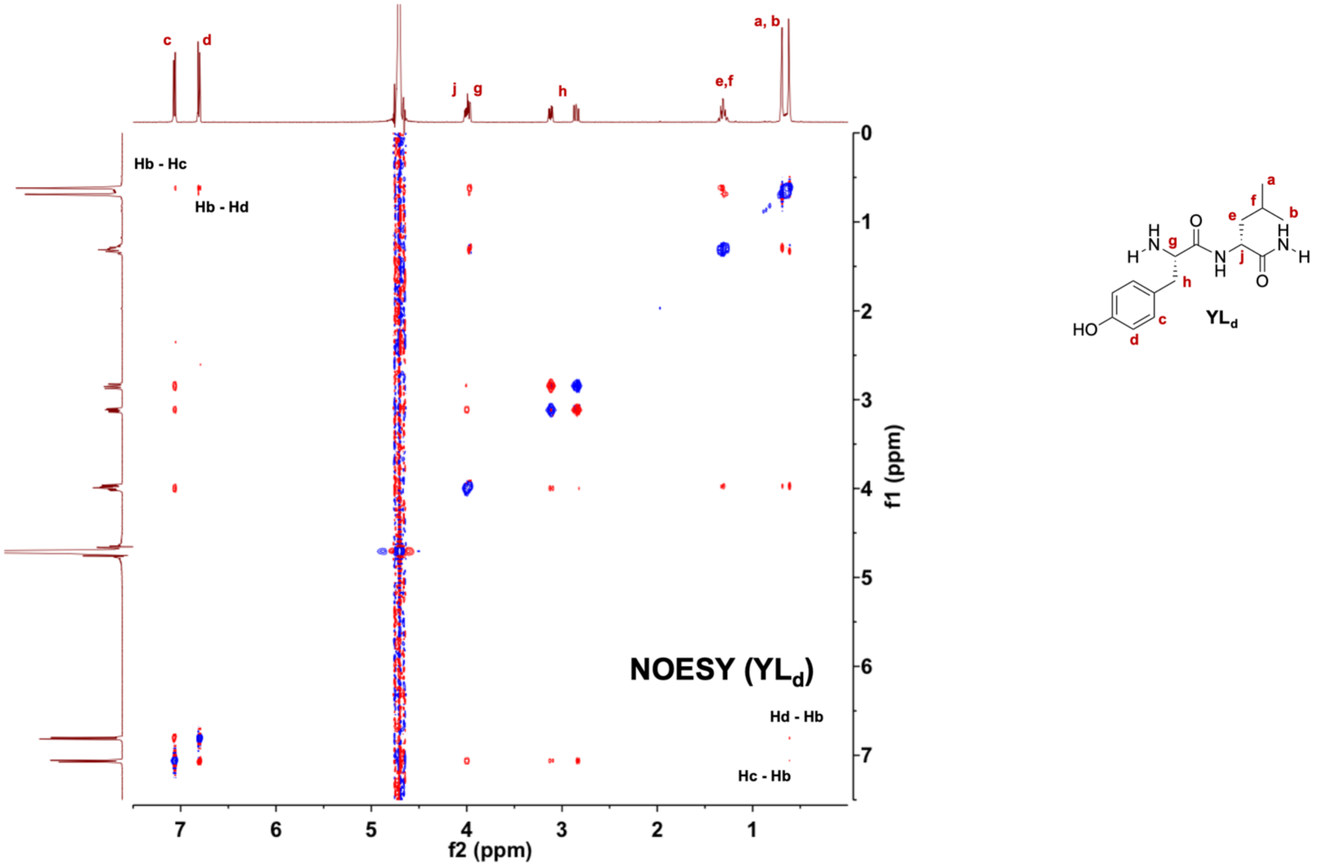

Figure S16. NOESY of YL$_d$. (500 MHz, 298 K, D_2O).

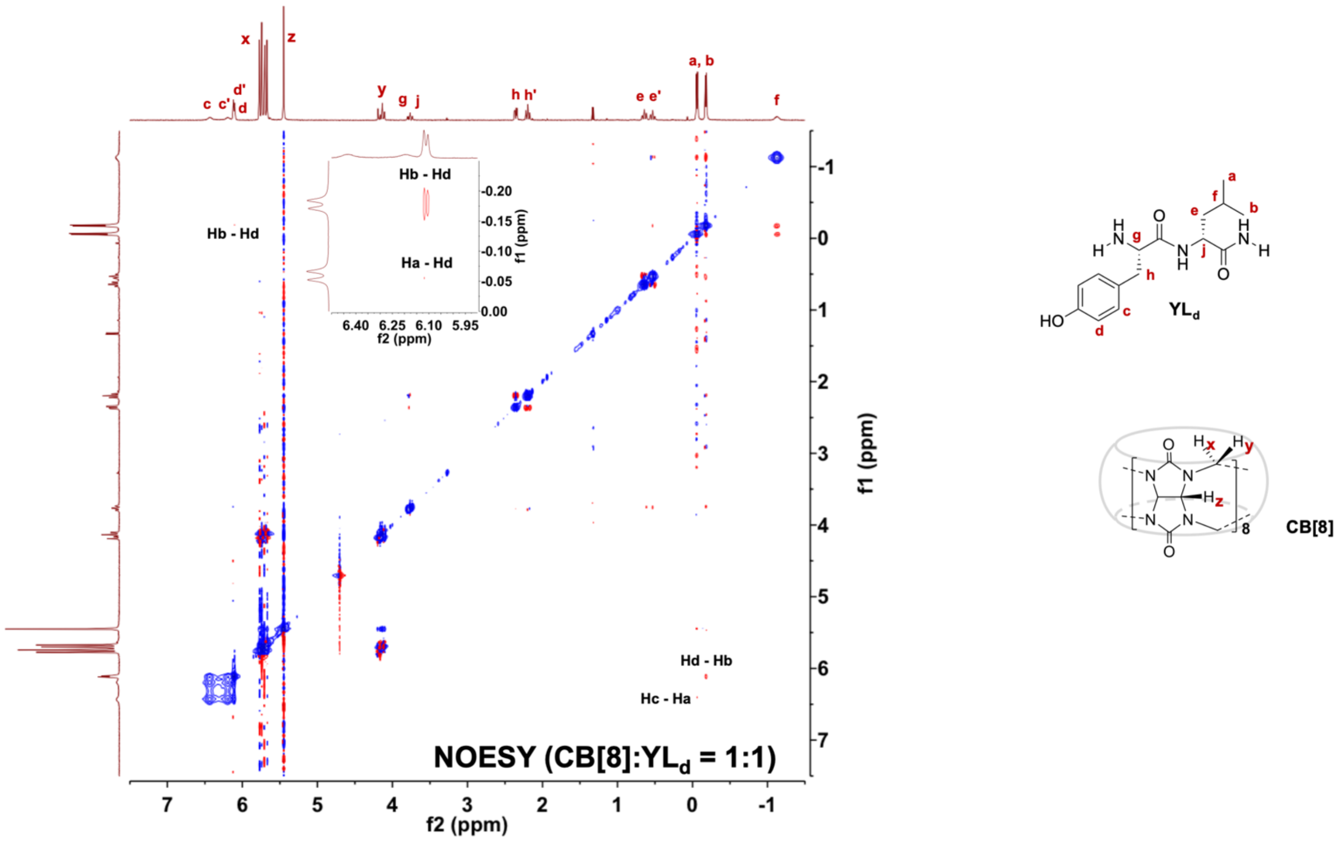

Figure S17. NOESY of CB[8]: YL$_d$ (1:1). (500 MHz, 298 K, D_2O).

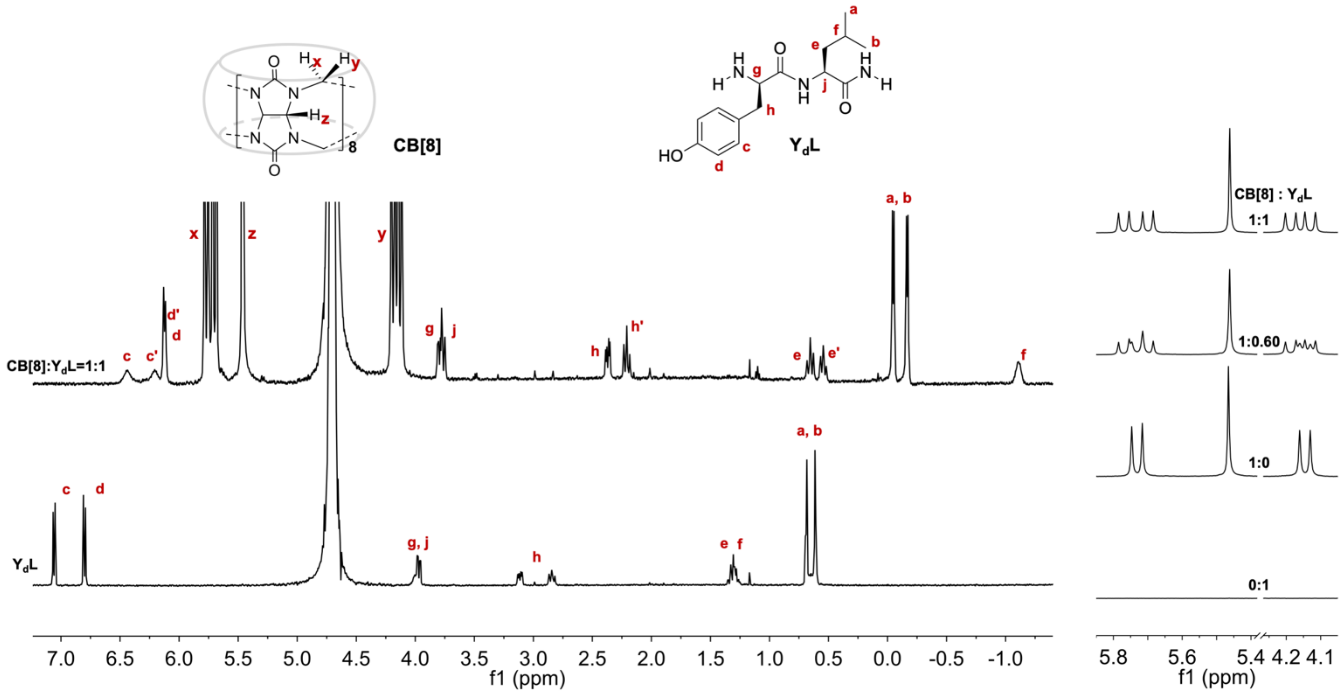

Figure S18. ^{1}H NMR of Y$_d$L titrated into CB[8] until a ratio of 1:1. (500 MHz, 298 K, D_2O).

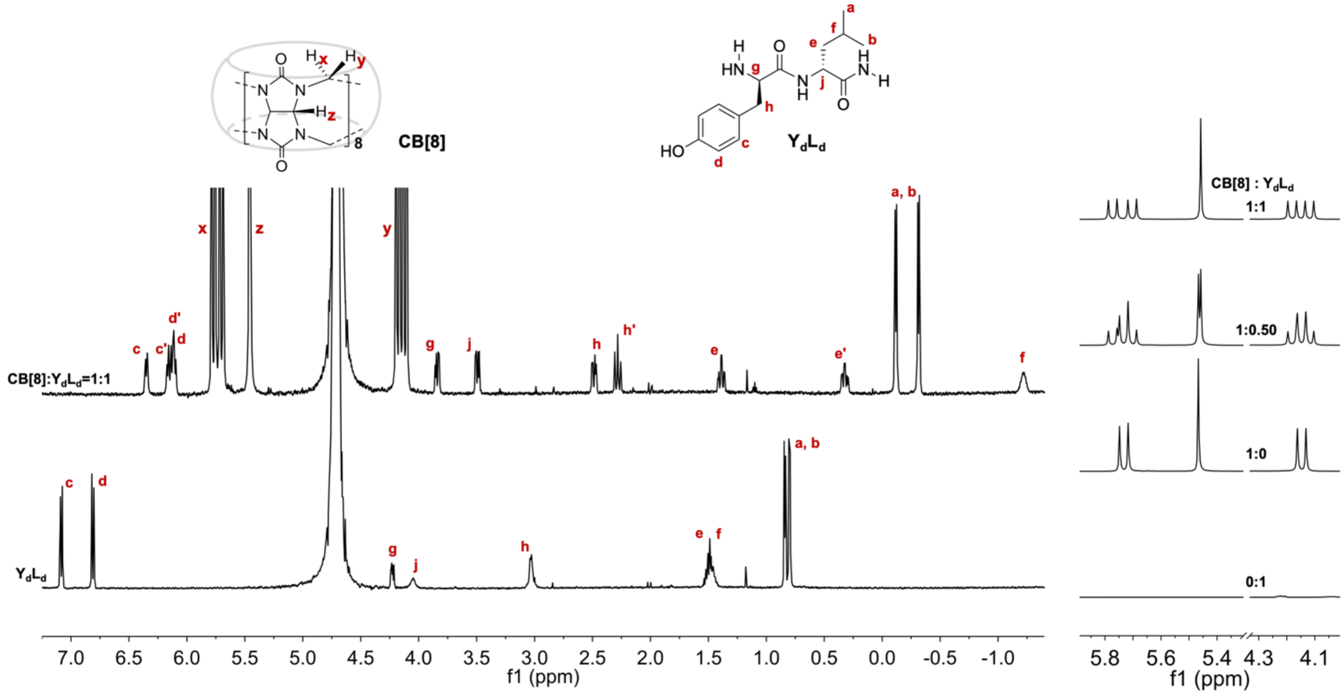

Figure S19. ^{1}H NMR of Y_dL_d titrated into CB[8] until a ratio of 1:1. (500 MHz, 298 K, D_2O).

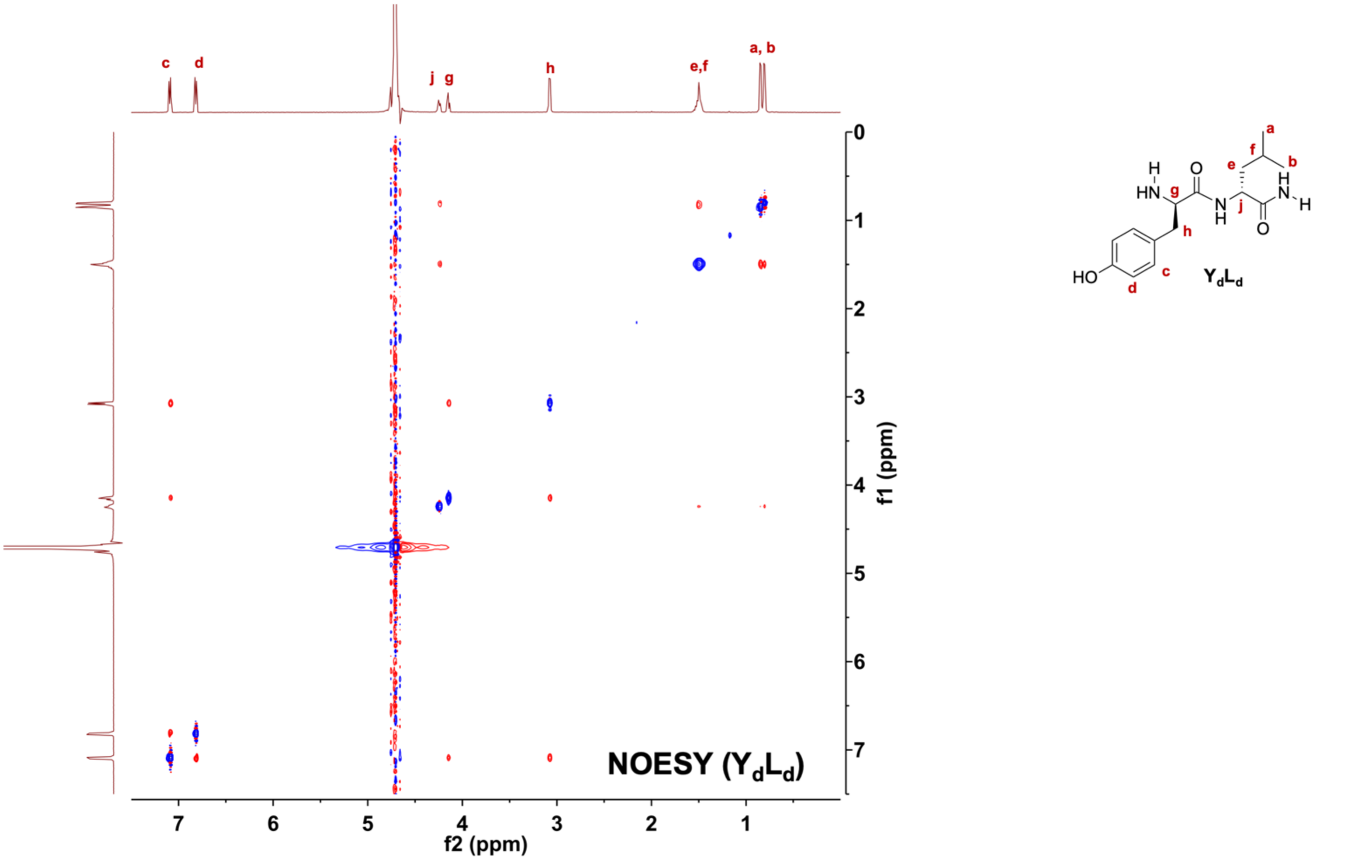

Figure S20. NOESY of Y_dL_d. (500 MHz, 298 K, D_2O).

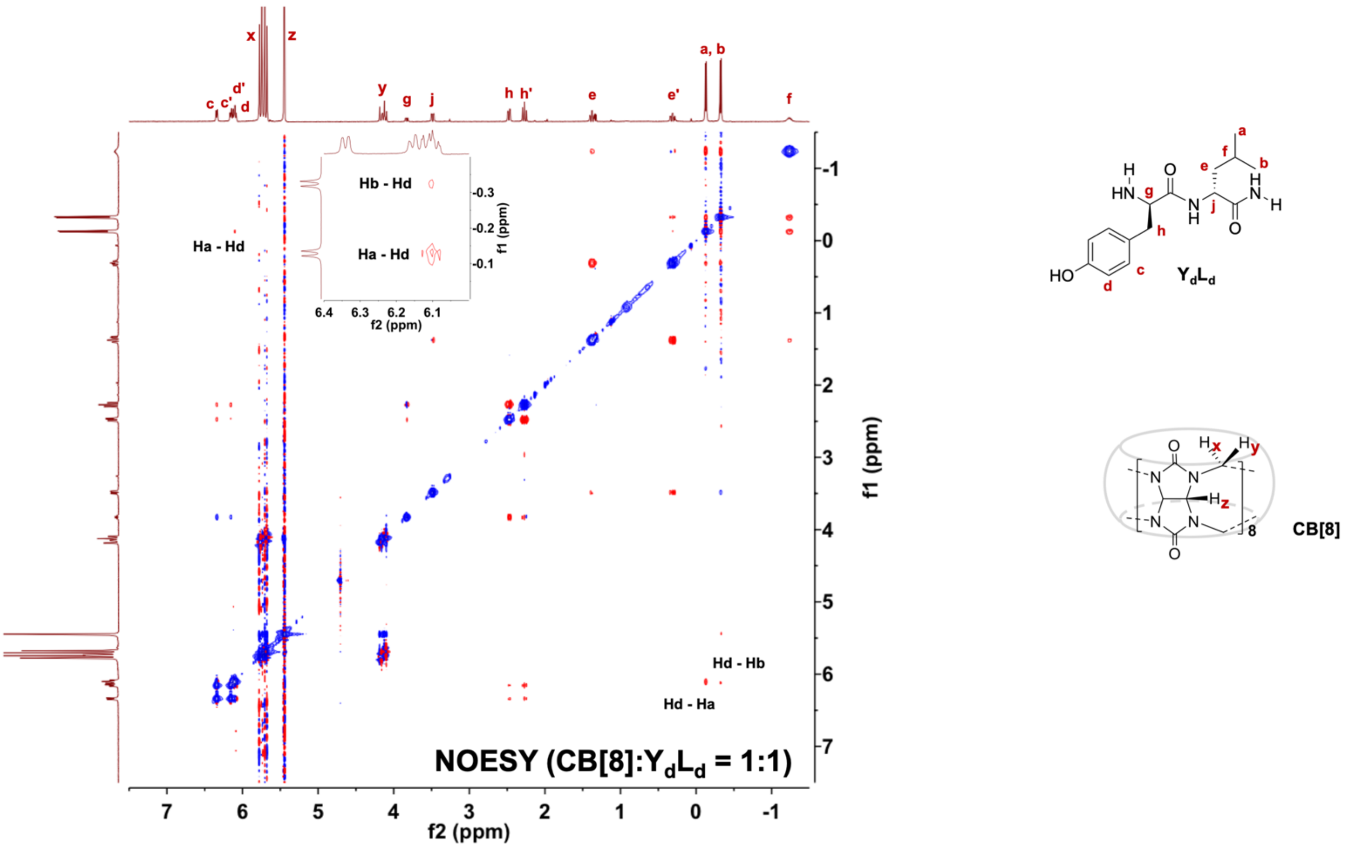

Figure S21. NOESY of CB[8]:Y_dL_d (1:1). (500 MHz, 298 K, D_2O).

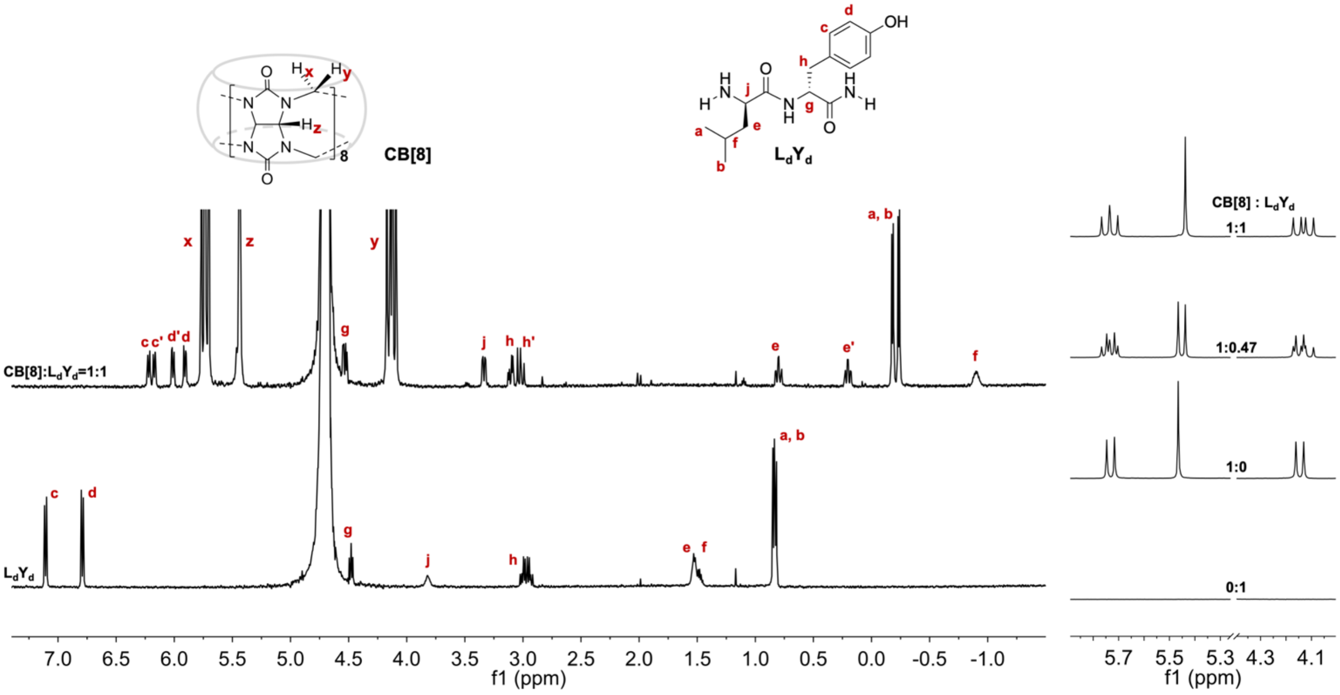

Figure S22. ^{1}H NMR of L_dY_d titrated into CB[8] until a ratio of 1:1. (500 MHz, 298 K, D_2O).

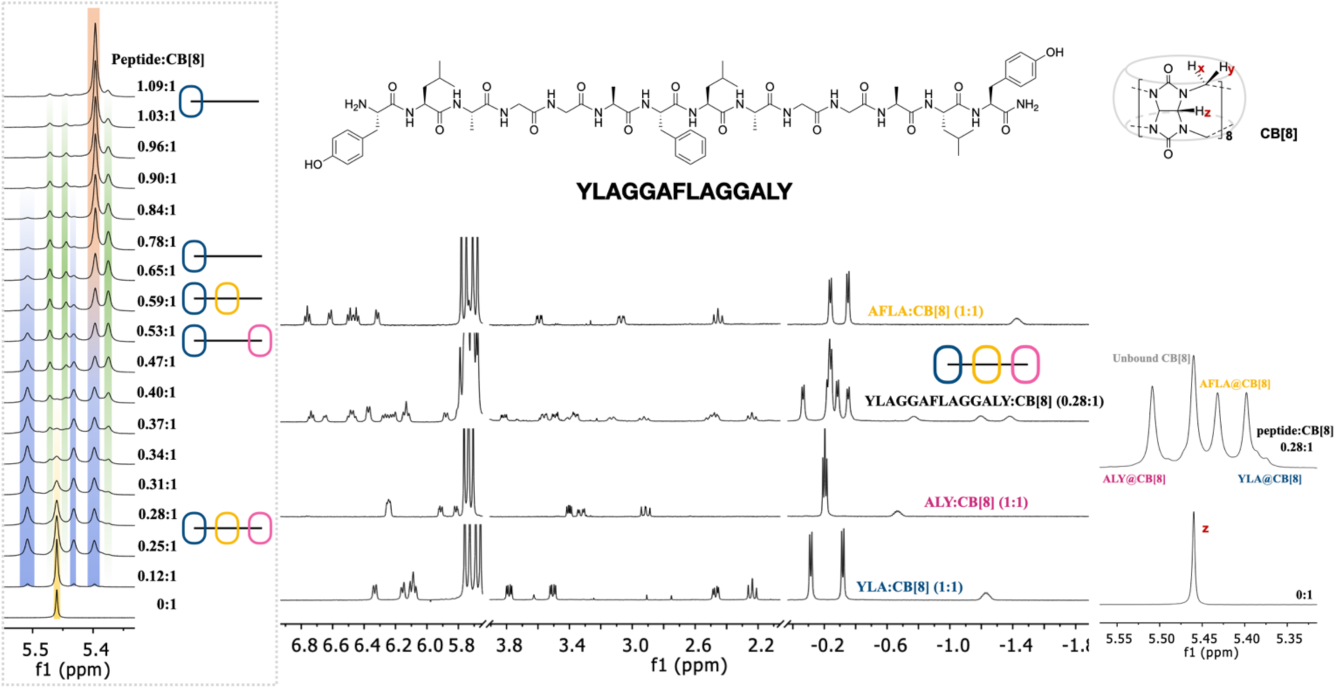

Figure S23. ^{1}H NMR of YLAGGAFLAGGALY titrated into CB[8] (left). ^{1}H NMR of AFLA@CB (1:1), ALY@CB[8] (1:1) and YLA@CB[8] (1:1), highlighting superimposed spectrum of YLAGGAFLAGGALY@CB[8] (0.28:1) (center and right). All spectra collected at 500 MHz, 298 K, D_2O.

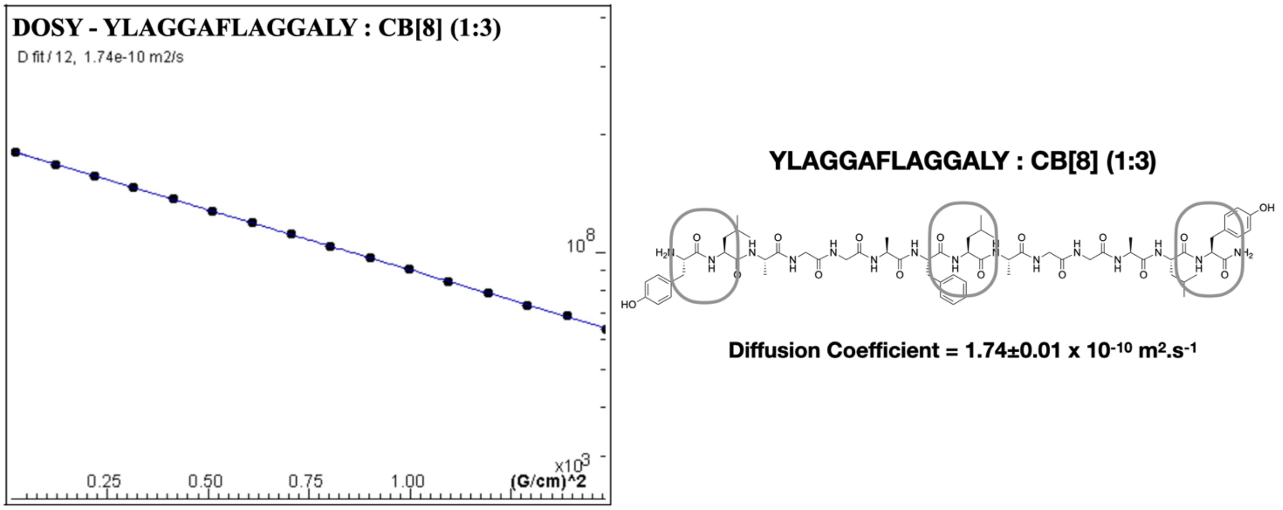

Figure S24. DOSY of YLAGGAFLAGGALY:CB[8] at a ratio of 1:3 (500 MHz, 298 K, D_2O).

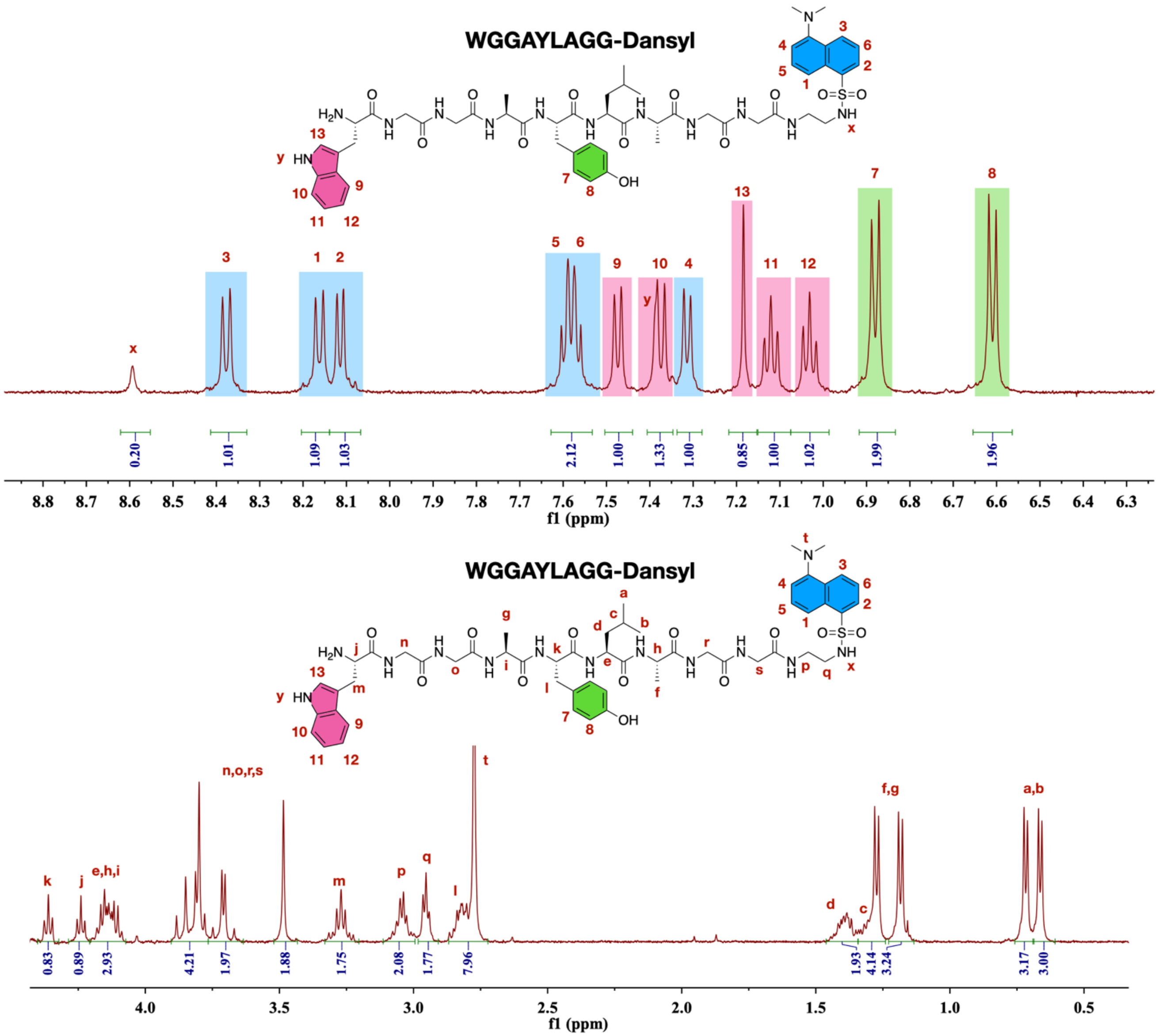

Figure S25. ^{1}H NMR of WGGAYLAGG-Dansyl (h-peptide) (500 MHz, 298 K, D_2O).

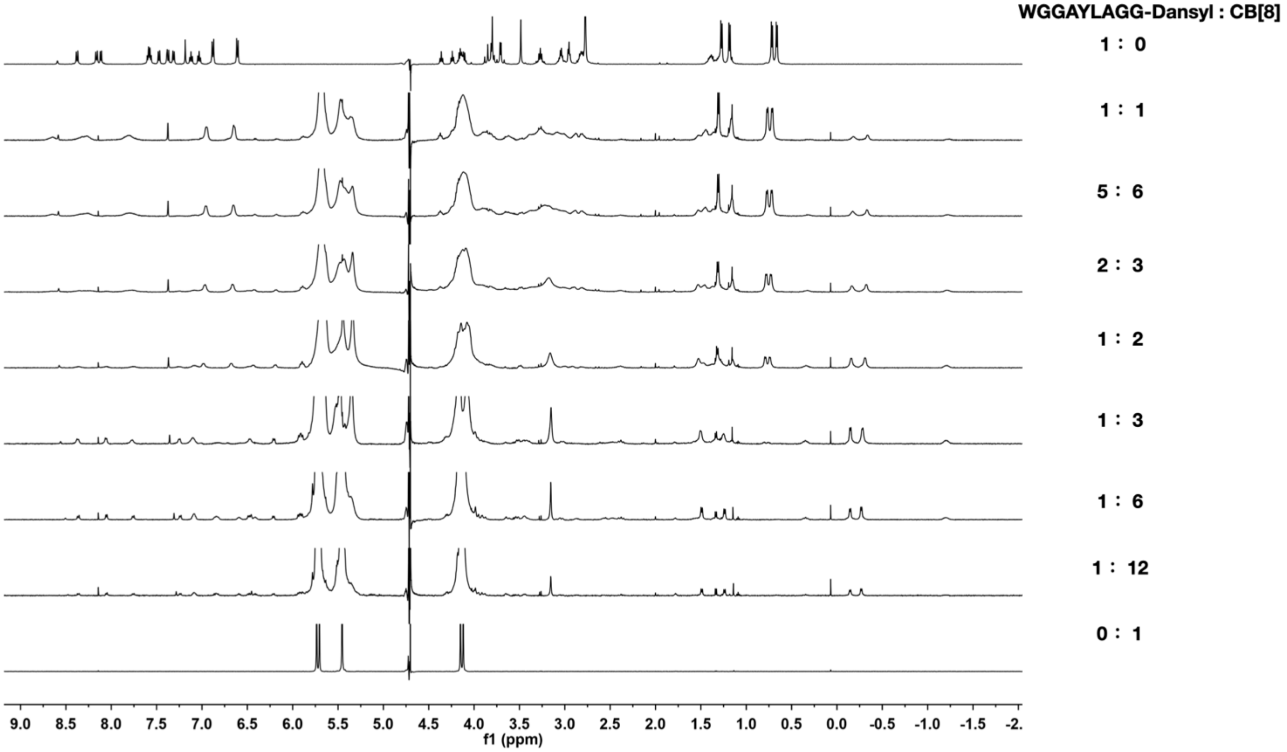

Figure S26. ^{1}H NMR of WGGAYLAGG-Dansyl (h-peptide) titrated into CB[8] until a ratio of 1:12. Broad peaks and indistinguishable binding modes are observed until CB[8] is in excess (500 MHz, 298 K, D_2O).

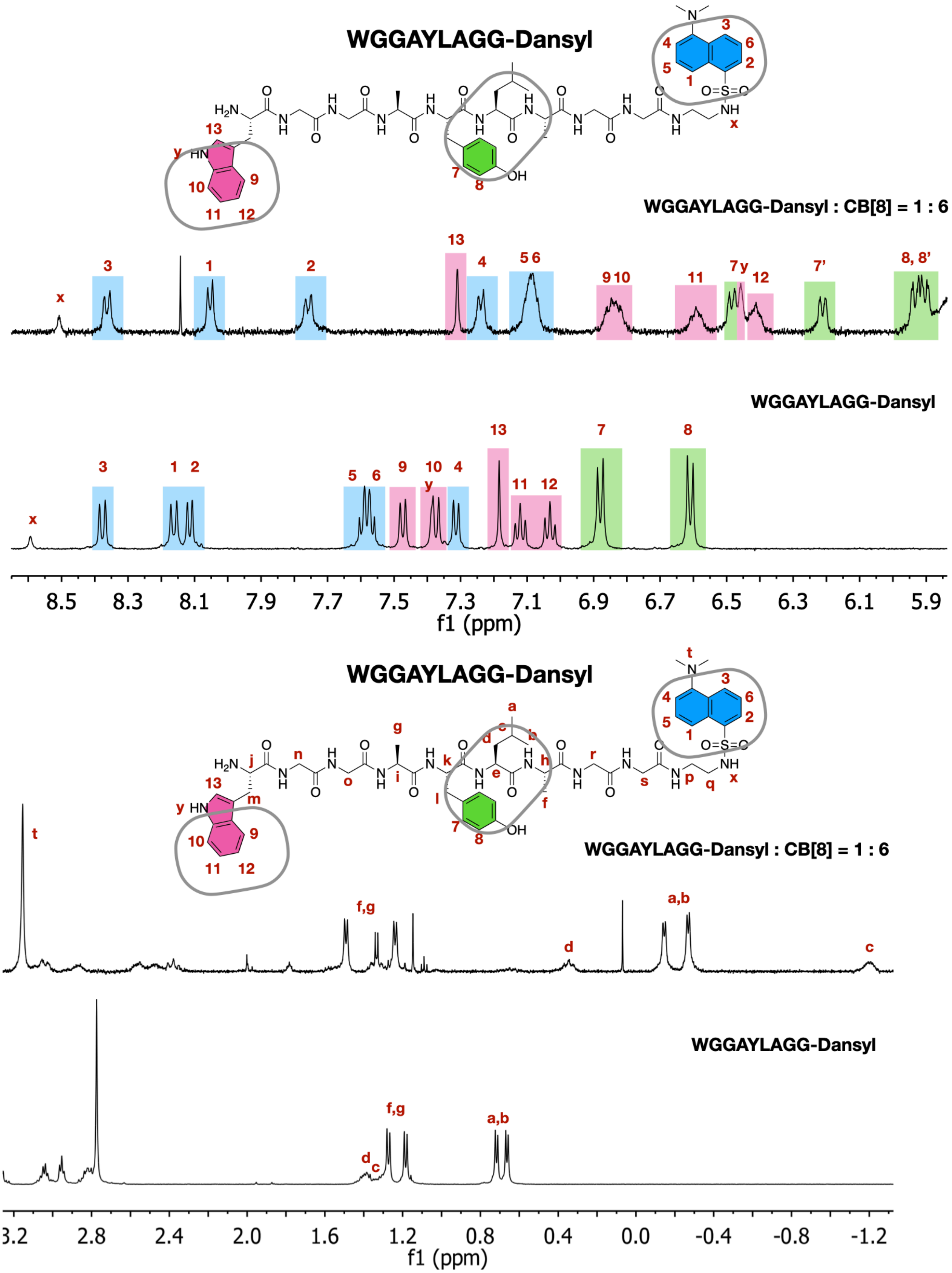

Figure S27. ^{1}H NMR of WGGAYLAGG-Dansyl (h-peptide):CB[8] at a ratio of 1:6 (500 MHz, 298 K, D_2O).

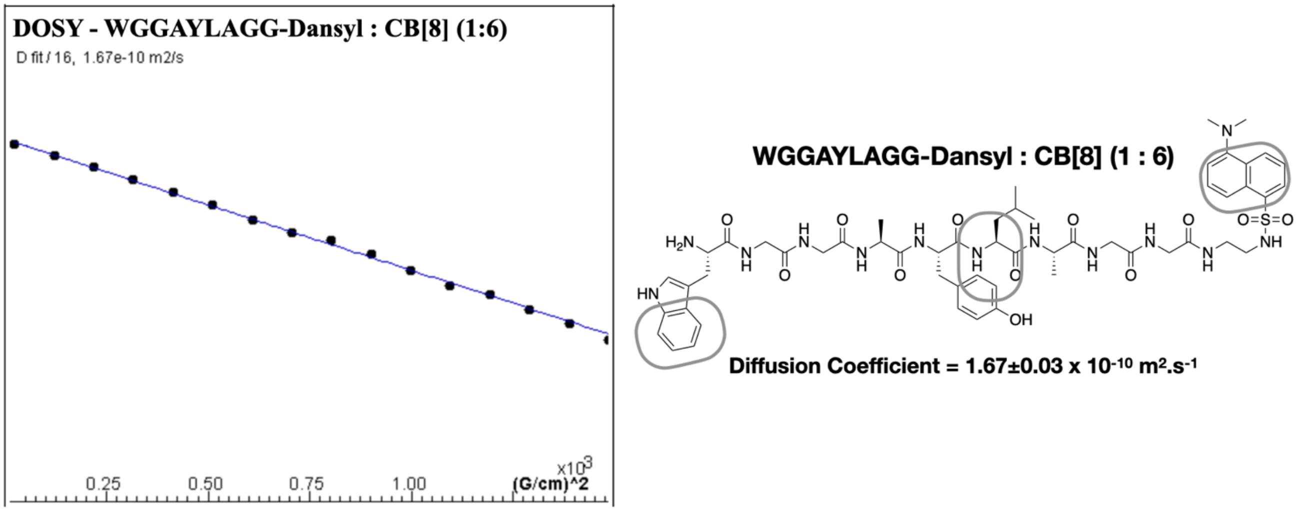

Figure S28. DOSY of WGGAYLAGG-Dansyl (h-peptide):CB[8] at a ratio of 1:6 (500 MHz, 298 K, D_2O).

Table S2. Diffusion Coefficients derived from DOSY for CB[8] 1:3 complexes

Species	Diffusion Coefficient (10^{-10} m^2. s^{-1})
Ant14Me:CB[8] (1:3) [4]	1.85±0.01
Ph135Me:CB[8] (1:3) [4]	1.79±0.01
YLAGGAFLAGGALY:CB[8] (1:3)	1.74±0.01
WGGAYLAGG-Dansyl:CB[8] (1:3)	1.67±0.03

SI-5 UV/Vis of h-peptide and its CB[8] complex

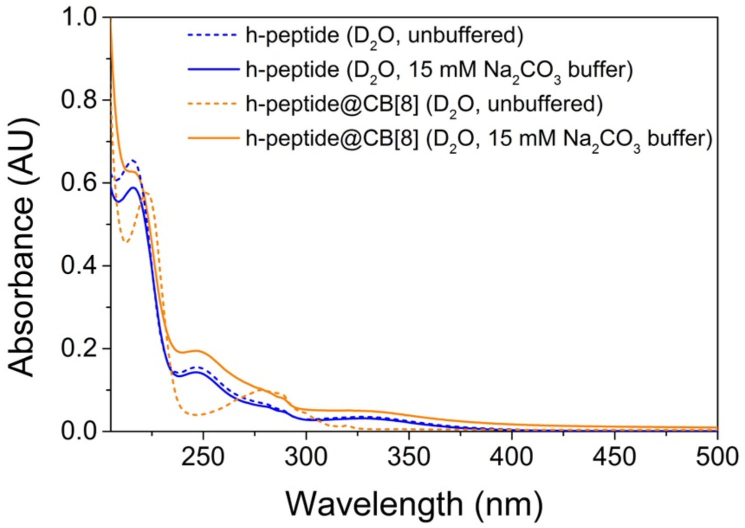

Figure S29. UV/Vis spectra of WGGAYLAGG-Dansyl (h-peptide) and its CB[8] complex (with excess CB[8], 1:6 h-peptide:CB[8]) in different buffers. All measurements were performed at a h-peptide concentration of 23 μM.

Reference:

[1] Day, A.; Arnold, A. P.; Blanch, R. J.; Snushall, B. *J. Org. Chem.* **2001**, *66* (24), 8094–8100.

[2] Brautigam, C. A.; Zhao, H.; Vargas, C.; Keller, S.; Schuck, P. *Nat. Protocols* **2016**, *11* (5), 882–894.

[3] Wu, G.; Clarke, D. E.; Wu, C.; Scherman, O. A. *Org. Biomol. Chem.* **2019**, 17, 3514–3520.

[4] G. Wu, I. Szabo, E. Rosta, O. A. Scherman, *Chem. Commun.* **2019**, *55*, 13227–13230.
